# Radionuclidic Impurity Pathways in Cyclotron Production: Insights from ^18^F and Emerging Theranostic Radionuclides

**DOI:** 10.3390/molecules31213987

**Published:** 2026-11-05

**Authors:** Teresa Jakubowska, Magdalena Długosz-Lisiecka, Jarosław Jankowski, Michał Biegała

**Affiliations:** 1 Department of Medical Imaging Technology, Medical University of Lodz, ul. Lindleya 6, 90-131 Łódź, Poland; 2 Department of Medical Physics, Copernicus Memorial Hospital in Lodz Comprehensive Cancer Center and Traumatology, Pabianicka 62, 93-513 Łódź, Poland; 3 Institute of Applied Radiation Chemistry, Faculty of Chemistry, Lodz University of Technology, Wróblewskiego 15, 90-924 Łódź, Poland; 4 Department of Nuclear Medicine, The Regional Specialist Hospital in Olsztyn, Żołnierska 18, 10-561 Olsztyn, Poland

**Keywords:** cyclotron production, theranostic radionuclides, fluorine-18, radionuclidic impurities, activation products, radiation protection

## Abstract

(1) Cyclotron production of medical radionuclides can generate unintended radionuclidic impurities, influencing product quality, radiation safety, and radioactive waste management. This review aims to establish a general mechanistic framework for the formation, transfer, and control of such impurities. (2) We identified published data on theranostic radionuclides, liquid- and solid-target production, ^18^F-associated impurities, and activation products through a Scopus search and reference screening. A total of 147 publications were qualitatively synthesized. (3) Production of ^18^F from H_2_^18^O served as a reference model for tracing impurity pathways from irradiation through transfer, purification, radiosynthesis, and waste management. Three principal sources can be distinguished: competing nuclear reactions involving the target isotope and residual isotopes; reactions involving other target-matrix constituents; and activation of target windows, bodies, and auxiliary materials. Impurity fate depends on release from the formation site, transfer into the processing stream, and retention or removal during purification and radiosynthesis. (4) The ^18^F model demonstrates that all of these stages should be considered when designing and validating new cyclotron-production routes. Co-produced isotopes of the product element require predominantly upstream control, whereas chemically distinct radionuclides can often be separated but are redistributed within the manufacturing system.

## 1. Introduction

Radiopharmaceuticals play a crucial role in the noninvasive imaging of metabolic and molecular processes, the localization and characterization of pathological lesions, the quantitative assessment of biodistribution and pharmacokinetics, treatment planning and monitoring, and the targeted delivery of ionizing radiation to specific tissues. Integrating diagnostic and therapeutic approaches into a unified clinical strategy forms the basis of theranostics, in which molecular imaging helps determine patient eligibility for targeted therapy, assess target expression, guide individualized activity prescription, and monitor treatment response [1,2,3,4].

Medical cyclotrons provide conventional short-lived positron emitters, such as ^18^F, ^11^C, ^13^N, and ^15^O, as well as an expanding range of metallic and therapeutic radionuclides [1,2,3,4]. Radionuclides with established or advanced clinical applications include ^18^F and ^68^Ga for routine PET imaging, ^64^Cu and ^89^Zr for applications requiring longer imaging windows, and ^111^In and ^99m^Tc for SPECT [5,6,7,8,9,10,11,12,13,14,15,16]. Direct cyclotron production of ^99m^Tc provides an alternative to the traditional ^99^Mo/^99m^Tc generator-based supply chain [9,10]. ^68^Ga is widely used in radiopharmaceuticals to target somatostatin receptors and PSMA [5,6,7,8]. ^64^Cu enables imaging over an extended time window and may serve as the diagnostic member of an element-matched pair with ^67^Cu [11,17]. The long half-life of ^89^Zr favours the labelling of antibodies and other slowly distributed macromolecules for immuno-PET [12,13,14,15].

Another group comprises radionuclides for which efficient production, purification, and radiolabelling methods have already been developed and for which the corresponding radiopharmaceuticals are being evaluated in translational or clinical studies. Representative examples include ^67^Cu for targeted β^−^ therapy [17], ^44^Sc as a PET emitter forming an element-matched theranostic pair with ^47^Sc [18,19,20,21,22,23,24], ^86^Y for imaging and dosimetry before ^90^Y therapy [25,26,27], and the ^203^Pb/^212^Pb pair, which combines SPECT imaging with therapy based on α-particle emission from the daughter radionuclides of ^212^Pb [28,29,30,31]. α-Emitters such as ^211^At and ^225^Ac have been evaluated in early clinical and translational studies [32,33,34]. Their development involves not only assessing biological efficacy but also scaling up cyclotron production, automating processing and synthesis, ensuring adequate radionuclidic and chemical purity, and establishing appropriate radiation-protection procedures [17,18,19,20,21,22,23,24,25,26,27,28,29,30,31,32,33,34,35].

In parallel, a large group of radionuclides remains at the preclinical or proof-of-concept stage. It includes ^61^Cu as a shorter-lived PET emitter in the ^61^Cu/^64^Cu/^67^Cu theranostic family [36,37]; ^43^Sc and ^47^Sc [21,22,23,24]; longer-lived positron emitters ^51^Mn and ^52^Mn [38,39,40]; the potential ^55^Co/^58m^Co pair [41,42,43]; and the terbium radionuclides ^152^Tb and ^155^Tb [44,45,46]. Radionuclides such as ^165^Er [47,48,49] and ^167^Tm [50] remain under development as therapeutic or dosimetric agents requiring complex lanthanide separation, whereas ^119^Sb [51,52,53] and ^135^La [54] are being investigated for Auger-electron therapy. Production platforms relevant to in vivo or ex vivo radionuclide-generator systems, including^103^Pd/^103m^Rh [55] and ^62^Zn/^62^Cu [56], are also under investigation. Similar efforts have been directed toward radiohalogens ^76^Br, ^77^Br, and ^80m^Br [57,58], as well as ^45^Ti [59]. Despite their diversity, these systems share several common objectives: establishing reproducible production methods that yield sufficient activity for biological studies, developing selective separation methods to isolate the desired radionuclide from target materials and radionuclidic byproducts, and demonstrating that the final radionuclide precursor is suitable for labelling molecular vectors [21,22,23,24,36,37,38,39,40,41,42,43,44,45,46,47,48,49,50,51,52,53,54,55,56,57,58,59,60]. Table 1 provides an overview of the theranostic radionuclides discussed in this review, their diagnostic or therapeutic roles, and representative cyclotron-production routes.

Among the theranostic systems under development, pairs based on lanthanum, barium, and bromine radionuclides occupy a distinctive position. The element-matched ^133^La/^135^La pair combines PET imaging with potential Auger-electron therapy; both radionuclides can be coproduced in a cyclotron from a natural barium target, while ^133^La can also be produced selectively from enriched ^134^Ba [54,61,62]. The radionuclides ^133^La and ^132^La are additionally being evaluated as diagnostic surrogates for ^225^Ac because the similarity of La^3+^ and Ac^3+^ chemistry enables the use of identical or closely related chelation systems. Similar biodistributions of [^132^La]La-NM600 and [^225^Ac]Ac-NM600 have been demonstrated in a preclinical model; however, the validity of this surrogate relationship requires pharmacokinetic concordance to be confirmed for each specific vector [63]. Another surrogate system pairs ^131^Ba with the α-emitters ^223^Ra and ^224^Ra: ^131^Ba is a SPECT radionuclide with chemistry similar to that of radium and may support delayed imaging, dosimetric planning, and treatment monitoring [64]. In contrast, the ^76^Br/^77^Br radiohalogen pair is fully element-matched; ^76^Br is used for PET, whereas ^77^Br is an Auger-electron emitter. ^80m^Br is also considered an alternative therapeutic. Production and separation methods developed for bromine radionuclides include the use of enriched CoSe targets, high-temperature dry distillation, and the recovery of ^76^Br and ^77^Br from parent krypton radionuclides captured in the off-gas stream of an irradiated aqueous system [57,58].

This broad spectrum of radionuclides demonstrates that contemporary cyclotron production is no longer confined to the routine manufacture of PET emitters. It spans a technological continuum: from radionuclides used daily in diagnostics through agents undergoing clinical investigation to experimental isotope pairs and surrogate systems [1,3,4]. Examples include ^133^La/^135^La, the ^132^/^133^La/^225^Ac and ^131^Ba/^223^Ra surrogate systems, and ^76^Br/^77^Br, for which optimal production reactions, target designs, separation procedures, and quality requirements are still being established [54,57,58,61,62,63,64]. Moreover, the increasing diversity of cyclotron-produced radionuclides increases the importance of controlling coproduced radionuclides and activation products because radiopharmaceutical quality depends not only on the activity yield of the desired radionuclide but also on its radionuclidic and chemical purity, molar activity, and compatibility with the subsequent radiolabelling process [1,2,3,4].

Maximizing activity at the end of bombardment is not equivalent to maximizing radiopharmaceutical quality. Increasing the beam energy or current may open competing reaction channels, increase the activation of windows, backings, and shielding, intensify radiolysis, and accelerate surface degradation [1,2,3,4]. The resulting byproducts may increase the patient dose, complicate the establishment of release specifications, and shorten the permissible shelf life [1,3,4]. At the same time, stable metals originating from the target, transfer line, or synthesis module may compete for the chelator, reduce the radiolabelling yield and molar activity, and increase batch-to-batch variability [65,66,67]. Therefore, radionuclide production and radiopharmaceutical manufacture should be considered parts of a single integrated process [1,2,3,4].

Table 1 shows that cyclotron production is technically feasible for many diagnostic and therapeutic radionuclides, including several element-matched theranostic systems based on Cu, Sc, Pb, La, and Br. However, the two members of a theranostic pair are not necessarily produced via the same technology. Whereas many diagnostic radionuclides can be obtained in medical cyclotrons, therapeutic partners such as ^90^Y, ^161^Tb, ^212^Pb, ^131^I, ^177^Lu, and radium radionuclides are still produced mainly in reactors or radionuclide generators. Theranostic development therefore frequently depends on integrating radionuclides originating from different production platforms while maintaining comparable chemical forms, radiolabelling performance, pharmaceutical quality, and dosimetric behaviour.

The table also distinguishes technical feasibility from routine availability. Some radionuclides, particularly ^149^Tb and ^152^Tb, require high-energy cyclotrons, complex target systems, multistep chemical or mass-separation procedures, and infrastructure available only at specialized centres. For this reason, the successful demonstration of a production route or the generation of measurable activity should be regarded as an important step in radionuclide development rather than as proof of clinical applicability. Before considering clinical implementation, production must also deliver reproducible activity yields, high radionuclidic and chemical purity, adequate molar activity, validated radiolabelling methods, reliable automation, and compliance with relevant quality and radiation protection requirements.

**Table 1:** Theranostic functions and cyclotron-production options for the radionuclides discussed in this review.

Theranostic Pair	Radionuclide	Diagnostic or Therapeutic Function	Cyclotron Production, Reaction, and Possible Target
^61^Cu/^67^Cu	^61^Cu	Diagnostic (PET). A positron emitter for imaging peptides and small molecules, including SSTR and PSMA ligands.	Yes. A solution of a natural zinc salt in a liquid target; alternatively, a solid ^62^Ni target via the ^62^Ni(p,2n)^61^Cu reaction [36,37,68].
	^67^Cu	Therapeutic (β^−^). Enables targeted radionuclide therapy; accompanying γ emission may support imaging and dosimetry.	Yes. A solid enriched ^70^Zn target; the ^70^Zn(p,α)^67^Cu reaction [17,69].
^64^Cu/^67^Cu	^64^Cu	Primarily diagnostic (PET), with therapeutic potential also arising from β^−^ and Auger-electron emission.	Yes. A liquid solution of enriched ^64^Ni irradiated with protons at approximately 16.9 MeV; alternatively, an electroplated solid ^64^Ni target via ^64^Ni(p,n)^64^Cu or a solid ^68^Zn target via ^68^Zn(p,nα)^64^Cu [11,70,71].
	^67^Cu	Therapeutic (β^−^). Use of the same element permits radiolabelling with the same chelator and vector as for ^64^Cu.	Yes. A solid enriched ^70^Zn target; the ^70^Zn(p,α)^67^Cu reaction [17,69].
^43^Sc/^47^Sc	^43^Sc	Diagnostic (PET). Used for quantitative imaging and may precede therapy with a ^47^Sc-labelled radiopharmaceutical.	Yes; under development. Solid targets of enriched ^43^Ca, such as CaCO_3_ or CaO; the ^43^Ca(p,n)^43^Sc reaction [72].
	^47^Sc	Therapeutic (β^−^), with the possibility of SPECT imaging owing to γ emission.	Yes. Foils or other solid V or Ti targets, including natV(p,x)^47^Sc and proton-induced reactions on Ti [22,23,24].
^44^Sc/^47^Sc	^44^Sc	Diagnostic (PET). Enables assessment of the biodistribution of a scandium radiopharmaceutical before ^47^Sc therapy.	Yes. A liquid natCa(NO_3_)_2_ solution in a syphon target via natCa(p,x)^44^Sc; for higher purity and yield, solid targets of enriched ^44^Ca, usually CaCO_3_ or CaO, are also used via ^44^Ca(p,n)^44^Sc [18,19,20,21,73].
	^47^Sc	Therapeutic (β^−^) and imaging (SPECT).	Yes. Solid Ti or V targets; selection of beam energy limits coproduction of other Sc isotopes [22,23,24].
^86^Y/^90^Y	^86^Y	Diagnostic (PET) and dosimetric. Enables prediction of biodistribution and absorbed dose before ^90^Y therapy.	Yes. A liquid Sr(NO_3_)_2_ solution in a nitrate medium or a solid enriched ^86^Sr target, such as SrCO_3_ or SrO, via ^86^Sr(p,n)^86^Y; Zr targets have also been investigated [25,26,27,74].
	^90^Y	Therapeutic (β^−^). Used, among other applications, in radioembolization and radioimmunotherapy.	Not routinely. Obtained mainly from a ^90^Sr/^90^Y generator rather than by direct cyclotron production [25,26,27].
^152^Tb/^161^Tb	^152^Tb	Diagnostic (PET). Its long half-life favours imaging of slowly distributing vectors.	Possible; specialized. Solid Gd or Gd_2_O_3_ targets; production may require higher-energy beams and complex lanthanide separation [44,45].
	^161^Tb	Therapeutic (β^−^, conversion electrons, and Auger electrons), particularly attractive for treating small lesions.	Not routinely. Most commonly produced in a reactor via ^160^Gd(n,γ)^161^Gd → ^161^Tb [44,45,46].
^155^Tb/^161^Tb	^155^Tb	Diagnostic (SPECT) and dosimetric. May be used for planning ^161^Tb therapy.	Yes. Solid targets of enriched Gd or Gd_2_O_3_; the ^155^Gd(p,n)^155^Tb and ^156^Gd(p,2n)^155^Tb reactions [44,45,46].
	^161^Tb	Therapeutic (β^−^ and low-energy electrons).	Not routinely. Mainly a reactor target of enriched ^160^Gd [44,45,46].
^152^Tb/^149^Tb or ^155^Tb/^149^Tb	^149^Tb	Therapeutic (α). The short range and high LET favour selective destruction of cancer cells.	Possible; specialized. High-energy reactions on lanthanide targets combined with multistep chemical separation or mass separation [60].
^203^Pb/^212^Pb	^203^Pb	Diagnostic (SPECT) and dosimetric. Enables prediction of the distribution of therapeutic ^212^Pb.	Yes. A solid enriched ^205^Tl target, for example electroplated or encapsulated; ^205^Tl(p,3n)^203^Pb or ^205^Tl(d,4n)^203^Pb [28,29,30,31].
	^212^Pb	Therapeutic, acting as an in vivo generator of the α-emitting daughter radionuclide ^212^Bi.	Not routinely produced by direct production. Most commonly obtained from generators based on ^224^Ra or ^228^Th [28,31].
^55^Co/^58m^Co	^55^Co	Diagnostic (PET). Enables assessment of the biodistribution of cobalt compounds.	Yes. Solid targets of enriched Fe or pressed ^58^Ni/Mg targets; the reaction and energy are selected to limit other Co isotopes [41,42,43].
	^58m^Co	Therapeutic (Auger electrons). Requires delivery of the radionuclide close to the cell nucleus or DNA.	Yes. A liquid Fe(NO_3_)_3_ solution in a 13 MeV syphon target, with concurrent formation of ^58^ᵍCo and ^56^Co; alternatively, solid targets of enriched Fe or Ni [42,43,75].
^124^I/^131^I	^124^I	Diagnostic (PET) and dosimetric, including imaging of iodine metabolism before ^131^I therapy.	Yes. A solid enriched tellurium target, such as Te or TeO_2_; the typical reaction is ^124^Te(p,n)^124^I, with iodine recovered by distillation [1].
	^131^I	Therapeutic (β^−^), with the possibility of γ imaging; used mainly for thyroid diseases.	Not routinely. Produced primarily in reactors from tellurium or as a fission product [1].
^68^Ga/^177^Lu	^68^Ga	Diagnostic (PET). Clinically used in SSTR- and PSMA-targeted radiopharmaceuticals for selecting patients for therapy.	Yes. ^68^Zn(p,n)^68^Ga: a liquid target containing a ^68^Zn salt in acidic solution or a solid target of pressed or electroplated ^68^Zn [5,6,7,8,76,77,78,79].
	^177^Lu	Therapeutic (β^−^), with the possibility of SPECT imaging; used, among other indications, in SSTR- and PSMA-targeted therapy.	Not routinely. Reactor production directly from ^176^Lu or indirectly via ^176^Yb [1].
^133^La/^135^La	^133^La	Diagnostic (PET). Low-energy positron emission favours high-resolution imaging, while the half-life permits studies lasting several hours.	Yes. A solid, sealed natural Ba target irradiated with approximately 22 MeV protons; alternatively, enriched ^134^BaCO_3_ via the ^134^Ba(p,2n)^133^La reaction [61,62].
	^135^La	Therapeutic (Auger electrons). The very short emission range requires localization of the radionuclide close to critical cellular structures.	Yes. Coproduced with ^133^La during irradiation of natural Ba; its proportion depends on beam energy and the isotopic composition of the target [54,61].
^132^La/^225^Ac	^132^La	Diagnostic (PET). A surrogate imaging radionuclide for assessing the biodistribution of ^225^Ac radiopharmaceuticals labelled to the same vector.	Yes. A metallic natural Ba target; proton-induced reactions at approximately 11.9 MeV. ^135^La is coproduced [63].
	^133^La	Diagnostic (PET). A chemical analogue of Ac^3+^, particularly useful for conjugates based on the macropa chelator.	Yes. An enriched ^134^BaCO_3_ target; the ^134^Ba(p,2n)^133^La reaction at approximately 22 MeV [62].
^132^/^133^La/^225^Ac	^225^Ac	Therapeutic (α). The lanthanum system is a surrogate system; concordance of biodistribution must be validated for the specific ligand and vector.	Yes. A sealed ^226^Ra target; the ^226^Ra(p,2n)^225^Ac reaction [34].
^131^Ba/^223^Ra or ^224^Ra	^131^Ba	Diagnostic (SPECT). A chemical analogue of radium for long-term imaging, dosimetric planning, and therapy monitoring.	Yes. A solid CsCl target; the ^133^Cs(p,3n)^131^Ba reaction at 27.5 MeV [64].
	^223^Ra/^224^Ra	Therapeutic (α). ^223^Ra is used clinically for skeletal lesions, whereas ^224^Ra is being developed, among other applications, for diffusing alpha-emitter radiation therapy (DaRT).	Not routinely. The therapeutic radionuclides were not produced by a cyclotron in this study; ^131^Ba serves as a diagnostic analogue [64].
^76^Br/^77^Br	^76^Br	Diagnostic (PET). Its long half-life permits transport and imaging of small molecules at late time points.	Yes. An enriched intermetallic Co^76^Se target; the ^76^Se(p,n)^76^Br reaction. Recovery through decay of ^76^Kr captured from the gas phase is also possible [57,58].
	^77^Br	Therapeutic (Auger electrons). Particularly attractive for labelling molecules that localize near DNA, such as PARP-1 inhibitors.	Yes. An enriched Co^77^Se target; the ^77^Se(p,n)^77^Br reaction. Alternatively, generated by decay of captured ^77^Kr [57,58].
^76^Br/^80m^Br	^80m^Br	Therapeutic (Auger and conversion electrons). Its short half-life favours use with rapidly distributing vectors.	Yes. An enriched Co^80^Se target; the ^80^Se(p,n)^80m^Br reaction [58].
Imaging radionuclide/^225^Ac	^225^Ac	Therapeutic (α). Its decay chain delivers several α particles and a very high local absorbed dose.	Yes. A solid, sealed ^226^Ra target, for example an electroplated target; the ^226^Ra(p,2n)^225^Ac reaction [34].
^89^Zr/therapeutic radionuclide labelling the same vector	^89^Zr	Diagnostic (immuno-PET). Its half-life is matched to antibody pharmacokinetics.	Yes. A liquid Y(NO_3_)_3_ solution in HNO_3_ or a solid Y target as a foil, coin, or deposited layer; the ^89^Y(p,n)^89^Zr reaction [12,13,14,15,74,80,81,82].

Note: the term “cyclotron production” encompasses both processes that can be performed with conventional medical cyclotrons and routes requiring specialized accelerators. Radionuclides designated “not routine” are currently obtained mainly from reactors or radionuclide generators.

### Radionuclidic Impurities: Definitions, Mobility, and Radiopharmaceutical Significance

The expanding range of radionuclides produced using cyclotrons is accompanied by the formation of by-product radionuclides that may become radionuclidic impurities. In this review, the term “radionuclidic impurity in the product” refers to any radionuclide other than the declared radionuclide that is present in the crude product, an intermediate, or the finished radiopharmaceutical. The term “activation product” has a broader scope, encompassing radionuclides formed in the target material, entrance window, target body, degrader, cyclotron components, or shielding, regardless of whether they are transferred into the product [1,2,3,4,83,84,85,86,87].

Radionuclidic impurities must be distinguished from radiochemical impurities, which are undesired chemical forms of the declared radionuclide, and from elemental impurities, including stable elements that may affect toxicity, radiolabelling yield, molar activity, or product stability. These categories require independent assessment, as high radiochemical purity does not necessarily indicate high radionuclidic purity or low levels of elemental contamination [1,2,3,4,65,66,67].

A further distinction can be made between mobile radionuclides, which may be released from their site of formation and enter process streams, and retained activation products, which remain associated with the irradiated material or structural component. This distinction is central to the present review because the formation of an activation product does not necessarily imply its presence in the radionuclide precursor or finished radiopharmaceutical [83,85,87,88,89].

The aim of this review is to systematically examine the mechanisms governing the formation, release, migration, separation, and control of radionuclidic impurities, as well as their effects on radionuclide precursor and radiopharmaceutical quality. The production of ^18^F from H_2_^18^O is used as the reference system, and the analysis is subsequently extended to emerging liquid- and solid-target production routes.

## 2. Review Methodology

The literature search was conducted in Scopus (accessed on 10 July 2026). Four search strategies were applied, covering therapeutic and theranostic radionuclides, production in liquid targets, ^18^F-related impurities, and a broader search addressing impurities, activation products, and cyclotron radionuclide production. The individual searches yielded 46, 48, 79, and 190 records, respectively, for a total of 363 records. After title normalization and the removal of 45 duplicates, 318 unique publications published between 2015 and 2026 remained.

Publications were considered eligible if they provided information relevant to the mechanistic interpretation of radionuclidic impurity formation, mobilization, transfer, separation, or control during cyclotron production. Eligible sources included experimental studies, modelling and computational investigations, technical and process-development reports, and review articles. Review articles were used primarily to identify production routes, establish terminology, and locate relevant primary publications. Findings concerning specific radionuclides, target and construction materials, irradiation parameters, and impurity pathways were based preferentially on primary studies.

Publications were excluded if they addressed only the clinical or biological applications of radiopharmaceuticals without relevant information on radionuclide production or radionuclidic purity. Studies focused exclusively on radiosynthesis or radiolabelling were also excluded unless they investigated impurities originating from radionuclide production. Additional exclusions concerned accelerator design or beam parameters without a demonstrated connection to activation or product quality, reactor-based production without a relevant accelerator-related component, and records for which the available information was insufficient to establish relevance to the objectives of this review. Publications for which the full text could not be accessed were also excluded when the available information was insufficient for a reliable mechanistic assessment.

Following title, abstract, and full-text screening, 223 records were excluded, and 95 publications were retained for synthesis. Details of the search strategies, including thematic areas, search terms, and numbers of retrieved records, are presented in Table 2. The complete Scopus search strategies, including search strings, Boolean operators, database filters, and publication-year limits, are provided in the “Methods and notes” worksheet of Supplementary Material S1. During full-text review and reference screening, an additional 52 publications not identified through the database searches were included because they provided relevant experimental evidence, methodological information, or background necessary for the mechanistic interpretation. Consequently, the final synthesis comprised 147 publications.

The review aimed to identify and synthesize recurring mechanisms of radionuclidic impurity formation, mobilization, transfer, separation, and control across different cyclotron-production technologies, rather than provide a detailed quantitative comparison of individual production methods. Although a more comprehensive assessment of each process stage and the completeness of reporting in individual studies would be valuable, such analyses would require separate, more narrowly focused reviews and would extend beyond the intended scope of this synthesis. Given the heterogeneity of the included publications, no formal study-level quality scoring was performed. Instead, limitations arising from incomplete reporting of irradiation conditions, target materials, analytical methods, and detection limits were considered when interpreting the available evidence.

**Figure 1:**
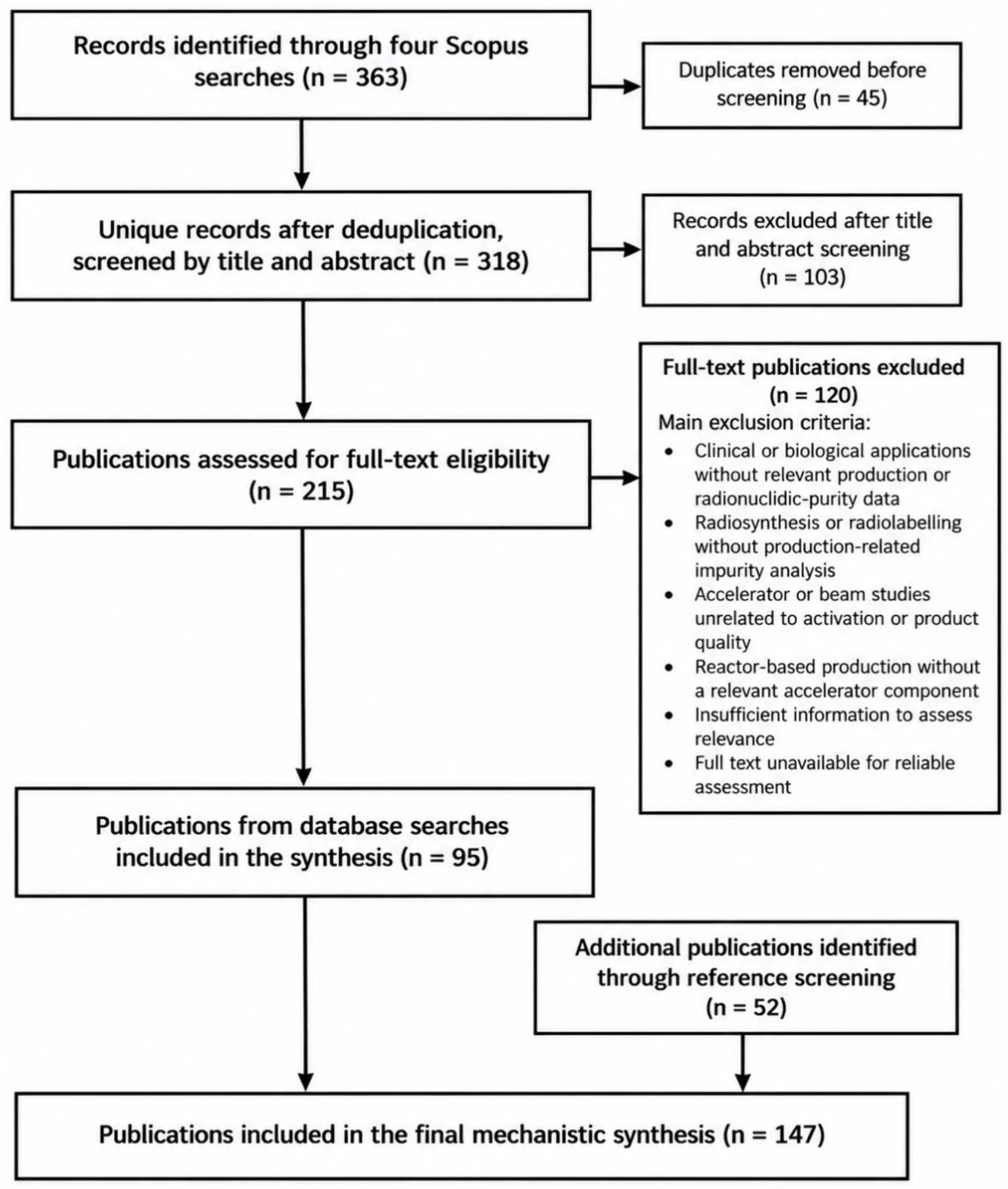
Flow diagram of publication selection, inspired by PRISMA 2020 and adapted to a scoping review with a mechanistic narrative synthesis.

Potential limitations include availability bias resulting from the exclusion of inaccessible full texts and the use of a single bibliographic database (Scopus), which precludes classification as a fully systematic review. To enhance transparency and reproducibility, selected PRISMA 2020 and PRISMA-ScR elements were adopted, including explicit reporting of search strategies, deduplication, selection procedures, exclusion reasons, and supplementary sources. The resulting flow diagram should therefore be regarded as PRISMA-inspired and adapted to the scoping nature of the review (Figure 1).

## 3. Production of ^18^F from H_2_^18^O as a Reference Model

The production of ^18^F via the ^18^O(p,n) ^18^F reaction is a particularly useful model because it combines reactions in a liquid material, direct activation of the window and target body, the contribution of secondary neutrons, the release of activation products, and their subsequent fate in the radiopharmaceutical process. For this radionuclide, the entire chain can be traced from the target, through transfer and the QMA cartridge, to [^18^F]FDG, [^18^F]NaF, or [^18^F]FMCH and to the waste streams. This makes it possible to distinguish radionuclides formed within the installation from those that actually reach the product released for use [83,84,85,86].

### 3.1. Target Assembly and Sources of Byproducts

A typical water target is a pressurized assembly that usually contains several millilitres of H_2_^18^O with an isotopic enrichment exceeding 95%. It comprises a target chamber, a thin entrance window separating the liquid from the vacuum of the beam line, a cooling system, filling and emptying ports, sealing components, and a line transporting the irradiated water to the synthesis module. The window is most commonly made of Havar, niobium-coated Havar, or niobium; some designs also include an additional vacuum foil. The target body is usually water cooled, whereas the window may be cooled by a helium flow. The exact volume, foil thickness, operating pressure, and geometry depend on the manufacturer and the permissible beam current [66,87,90,91].

**Figure 2:**
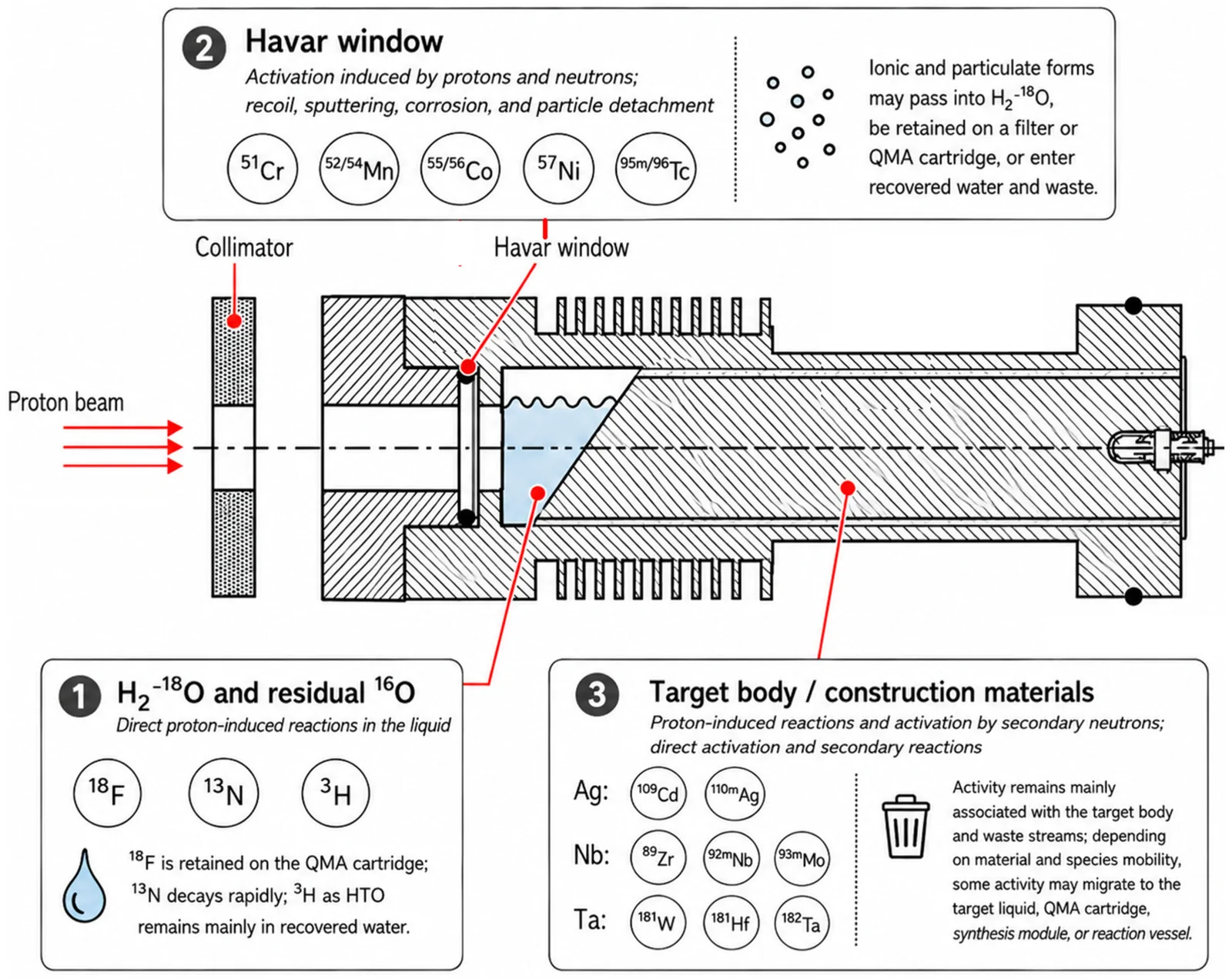
Sources, formation mechanisms, and typical fates of radionuclidic impurities during the production of ^18^F from H_2_^18^O.

Havars provide high mechanical strength; however, their composition (mainly Co, Cr, Fe, and Ni, with smaller fractions of W, Mo, and Mn) enables numerous proton- and neutron-induced activation reactions. A silver target body predominantly produces Ag- and Cd-derived radionuclides, whereas Nb and Ta reduce, but do not eliminate, activation products. During irradiation, protons lose energy in the entrance window and the H_2_^18^O target solution, and a fraction of the beam may interact with downstream components. In addition, the ^18^O(p,n)^18^F reaction generates secondary neutrons that contribute to activation outside the target volume. Consequently, impurity formation should be considered across the entire system, including the entrance foil, target solution, chamber walls and seals, transfer lines, and synthesis module (Figure 2) [84,85,86,87,90,91,92].

### 3.2. Products Formed Directly in the Water

In addition to ^18^F, ^13^N may be formed in the water from residual ^16^O via the ^16^O(p,α)^13^N reaction. Because of its approximately 10 min half-life, the contribution of ^13^N decreases rapidly during transfer and synthesis [93,94]. In contrast, ^3^H, which can be formed via the ^18^O(p,t)^16^O reaction, behaves differently. Tritium is expected to occur predominantly in water-associated forms, including HTO; it remains in the liquid phase and is not removed by columns designed to capture metal ions or fluoride. In routine production, its activity is correlated with the proton fluence and ^18^F activity [95,96].

The example of ^3^H demonstrates that “purification” of the product and “removal” of activity from the process are not synonymous. Tritium may be undetectable in the final radiopharmaceutical while remaining in the recovered H_2_^18^O and requiring a separate activity balance over repeated recycling cycles. Conventional distillation efficiently reduces many metallic radionuclides and organic impurities but does not provide selective, complete separation of HTO from enriched water [97,98,99,100].

### 3.3. Activation of the Havar Window (Foil) and Target Body

In Havar, (p,n), (p,2n), (p, np), (p,α), and secondary reactions produce, among others, ^51^Cr, ^52^/^54^Mn, ^55^Fe, ^55^/^56^/^57^/^58^Co, ^57^Ni, ^59^Ni, ^95m^/^95^/^96^Tc, and Re radionuclides such as ^184^Re. In silver, ^109^Cd forms in a proton-induced reaction, and ^110m^Ag associated with secondary neutron activation has been observed, among other products. Niobium systems have yielded ^89^Zr, ^92m^Nb, and ^93m^Mo, whereas tantalum systems have yielded ^181^W, ^181^Hf, and ^182^Ta. Depending on the target design and the chemical form of the products in the water or on the QMA cartridge, ^7^Be and ^65^Zn have also been identified [83,85,86,87,92,94,101,102,103].

Beam energy changes the accessibility of reaction channels, but reducing the energy does not eliminate activation. Products originating from Havar and silver have also been observed at 9.6–11 MeV. Increasing the integrated charge increased the activity of ^56^Co and ^110m^Ag, although the relationship was not always proportional to the increase in ^18^F activity. This finding is consistent with the contributions of thermal processes and surface changes but does not demonstrate a specific corrosion mechanism. Activities should be compared after correction to a common reference time, preferably the end of bombardment (EOB) [94,101,104]. Calculations for an H_2_^18^O target irradiated with 13-MeV protons predicted the simultaneous formation of Havar activation products, including ^96^Tc, ^55^Fe, ^59^Ni, ^56^Co, ^52^Mn, ^186^Re, and ^58^Cu, further confirming the dependence of the impurity profile on beam energy and window composition [105].

### 3.4. Release: Recoil, Sputtering, Corrosion, Erosion, and Particulate Matter

A radionuclide formed in a foil or target body does not automatically become an impurity in the liquid. It must leave the surface layer through nuclear recoil, sputtering, radiation damage, corrosion, thermal erosion, or particle detachment. SRIM calculations revealed a concentration of displacement and recoil atoms near the water-contacting surface and particularly high mobility of the Co, Cr, Fe, and Ni constituents. The absolute value of proton energy loss in the foil reported in that study was not used in the present synthesis because its order of magnitude requires independent verification [103].

Irradiation is accompanied by water radiolysis, which generates reactive radicals and peroxides, as well as by localized thermal loading of the surface. An increasing temperature, gas-bubble formation, cyclic pressure changes, thermal stresses, and window ageing may alter the passive layer of the metal and facilitate the release of ions and particles. These mechanisms are a plausible link between material activation and the appearance of an activation product in the liquid; however, identification of the dominant process requires direct surface analyses and should not be inferred solely from correlations of activity with beam current or proton dose [87,103,104].

The presence of a particulate fraction is supported by filtration measurements: a proportion of the Co, Mn, and Cr radionuclides occurred in particles larger than several micrometres and filters positioned downstream of the target retained activation products. The niobium coating of Havar reduced the release of Co, Ni, Mn, Cr, Fe, and Mo, although it did not eliminate them completely. The highest impurity concentrations were often observed during the first cycles with a new window; the authors attributed this to the removal of surface contaminants and gradual conditioning, although the passivation mechanism was not directly demonstrated [83,85,87,93,103].

### 3.5. QMA, Radiosynthesis, and Redistribution of Activity

After EOB, the irradiated water is transferred to the module. The QMA cartridge retains [^18^F]F^−^, whereas H_2_^18^O passes into the recovery vessel. The behaviour of other radionuclides depends on their chemical and physical forms. More than 90% of the ^51^Cr and ^7^Be present in the water was retained on the QMA cartridge, whereas a substantial fraction of Co, Mn, and Ni passed through the water. The Cr, Mn, Co, and Be radionuclides retained on QMA were only weakly desorbed during fluoride elution, reducing their transfer to the reactor by approximately 30- to 100-fold. Nb, Ta, Re, and Tc radioisotopes behaved differently: they could be quantitatively sorbed on the anion exchanger and subsequently eluted to a large extent together with [^18^F]fluoride—under some conditions, up to approximately 80% of their activity. QMA is therefore a selective separation step but not a universal barrier [83].

Filtration provides a complementary barrier. In the samples examined, approximately 30% of the Co and Mn radionuclides and approximately 7% of the Cr were present in particles larger than 5 µm; consequently, a transfer-line filter may reduce the particulate fraction but does not remove ions or fine colloids. An additional cation-exchange cartridge can retain both radioactive and stable metal cations. During [^18^F]AlF-FAPI-74 labelling, the removal of cations originating from the target solution restored the expected radiochemical yield, as confirmed by ICP–MS analysis. This example concerns primarily elemental impurities but demonstrates the importance of multibarrier purification before synthesis [65,83].

During [^18^F]FDG synthesis, successive columns and sorbent beds retain unreacted precursors, free fluoride, organic products, and a proportion of the metals. From the perspective of batch release, these operations simultaneously serve to achieve adequate radiochemical purity, reduce precursor residues, and control impurities originating from the target. Final filtration through a validated membrane with a nominal pore size of 0.22 µm is part of the aseptic process and removes particles and retained microorganisms but does not remove dissolved ions or radionuclides. Process effectiveness must be evaluated together with filter integrity, aseptic conditions, and testing of the final product [85,88,106].

A similar effect has been demonstrated for [^18^F]NaF and [^18^F]FMCH. Following QMA processing and formulation, [^18^F]NaF exhibited very high radionuclidic purity, and no radionuclide impurity was detected in the finished [^18^F]FMCH above the method-specific detection limit [99,107,108,109]. Stage-by-stage measurements revealed that most impurity activity was retained on the QMA cartridge or transferred to liquid waste streams rather than to the released product [88,89]. Thus, a properly designed process can meet product requirements despite target activation, but the activity is redistributed to the recovered water, columns, cassettes, and waste solutions.

Recovered H_2_^18^O requires a separate management strategy. Distillation reduced the concentrations of ^51^Cr, ^54^Mn, ^56^/^57^/^58^Co, and ^65^Zn, as well as solvent residues [98]. A procedure combining coprecipitation, cocrystallization, and distillation removed approximately 99.7% of the radionuclides, 99.8% of the trace metals, and 99.5% of the organic compounds while enabling recovery of the costly enriched water [100]. Recovered water may nevertheless require radiological characterization and classification before reuse or disposal [93]. These processes do not, however, resolve the problem of HTO, whose physicochemical properties are similar to those of water [96].

In addition to byproduct radionuclides, the material of the transfer line also affects radiopharmaceutical quality. The transport of irradiated water through fluoropolymer tubing, such as PTFE or ETFE, increases the stable fluoride content relative to that of nonfluorinated tubing, which may reduce the molar activity of the ^18^F-labelled compounds. This is not a radionuclidic impurity, but it is an important example of the relationship between the installation design and the quality of the final radiopharmaceutical [66].

### 3.6. Conclusions from the ^18^F Model

The ^18^F production model demonstrates that the presence of radionuclidic impurities in the final product is governed by four consecutive stages: formation of by-product radionuclides; their mobilization and transfer into the crude post-irradiation fraction; separation and retention during purification and radiosynthesis; and final distribution between product and non-product fractions. While the relative contribution of individual mechanisms within these stages may vary among radionuclides, the framework remains broadly applicable to novel production systems when radionuclide-specific chemical behaviour, physicochemical properties, and product specifications are taken into consideration.

The common mechanisms observed in this model are further examined in the following sections for radionuclides produced in liquid and solid targets.

## 4. Novel Radionuclides Produced in Liquid Targets

Liquid-target production extends beyond the aqueous H_2_^18^O system routinely used for ^18^F production and encompasses chemically and technically diverse target platforms. Their common feature is that the irradiated material is present in a liquid phase that can be remotely introduced into and removed from the target chamber and subsequently transferred directly to a purification module. This facilitates automation, reduces personnel exposure, and allows existing medical cyclotron infrastructure to be adapted to the production of radiometals and other radionuclides without a dedicated solid-target station. These advantages are accompanied by limitations that are less important, or absent, in solid targets, including lower atomic density, solvent radiolysis, pressure build-up, possible precipitation, and the need to separate a trace quantity of the radionuclide product from a macroscopic quantity of target material [2,4].

### 4.1. Diversity of Liquid-Target Platforms

The most widely investigated systems are aqueous solutions of metal salts, particularly concentrated nitrate solutions under acidic conditions. In these targets, the solution serves simultaneously as the irradiated target medium, coolant, and chemical environment in which the product and byproducts are generated. The most mature example is ^68^Ga production by proton irradiation of [^68^Zn]Zn(NO_3_)_2_ in HNO_3_. This system demonstrates that target performance cannot be described by target concentration or beam current alone. The Zn concentration, HNO_3_ concentration, beam current, and irradiation time are coupled parameters because they simultaneously affect the number of target atoms, radiolysis, solubility, and thermohydraulic stability [5,8,76]. Similar nitrate-based systems have been applied to concentrated Sr(NO_3_)_2_ solutions for ^86^Y and Y(NO_3_)_3_ in HNO_3_ for ^89^Zr production. In the latter case, an elongated water-cooled chamber with dual helium-cooled foils was used to accommodate irradiation of the concentrated yttrium solution at relatively high current [74,81,82]. Thus, even within the apparently homogeneous group of nitrate-based liquid targets, the chemical matrix and target geometry are adjusted to the physicochemical properties of the target material and the required irradiation conditions.

Syphon-style liquid targets represent a distinct engineering solution developed primarily to improve target filling, liquid distribution, and pressure management during irradiation. A _nat_Ca(NO_3_)_2_ solution used for ^44^Sc production illustrates the importance of target geometry: the syphon design limited pressure build-up and enabled an increase in the applicable beam current, thereby increasing production capacity [73]. Concentrated Fe(NO_3_)_3_ solutions have also been irradiated in a syphon target for ^58m^Co production. In this case, the technological feasibility of irradiation and chemical recovery was accompanied by a different limitation, namely the simultaneous formation of ^58g^Co and ^56^Co. Because these radionuclides are isotopes of the product element, chromatographic removal of Fe does not resolve the radionuclidic-purity problem [75]. The syphon configuration therefore illustrates an important distinction between engineering control and radionuclidic control: improvements in pressure management and target robustness may increase the usable beam current, but they do not prevent competing nuclear reactions within the target material.

Another approach is the use of highly concentrated solutions of target compounds whose chemical form is selected specifically to increase the achievable concentration while maintaining stability under irradiation. Phosphomolybdic acid, H_3_(Mo_12_PO_40_)·12H_2_O, used for ^94m^Tc production is an important example. In an adapted standard PETtrace liquid target, phosphomolybdic acid permitted a substantially higher Mo concentration than simpler salts while remaining stable during proton irradiation. No visible degradation of the niobium target body or foil was observed under the conditions studied, and the product could be separated from the macroscopic Mo-containing matrix using an aqueous biphasic extraction resin [110]. However, the use of naturally abundant Mo simultaneously opens production channels leading to other Tc radionuclides. These isotopes follow ^94m^Tc through chemical separation and therefore cannot be removed by improving resin selectivity. This system demonstrates that optimization of the chemical form of the target may solve problems of concentration and matrix stability without eliminating radionuclidic impurities originating from the isotopic composition of the irradiated element.

Isotopic enrichment is consequently a cross-cutting variable rather than a separate target configuration. It determines which competing reaction channels are accessible and is particularly important when the byproducts are isotopes of the same element as the desired radionuclide. This is illustrated by liquid Zn targets for ^61^Cu, where isotopically enriched material provided higher radionuclidic purity than natural Zn, and by ^64^Cu production using an enriched ^64^Ni solution. In the latter case, post-EOB processing must remove a macroscopic quantity of Ni as well as Co, Fe, and Zn contaminants, whereas coproduced Cu isotopes remain in the product fraction [36,37,70,71]. The same principle applies to ^68^Ga, for which ^66^Ga and ^67^Ga accompany ^68^Ga during capture, elution, and radiolabelling, and to ^86^Y, for which ^85^Y and ^85m^Y cannot be separated chemically from the desired radionuclide. Therefore, isotopic enrichment, incident and exit energies, irradiation time, and cooling time are upstream variables that determine a component of radionuclidic purity that cannot subsequently be corrected by downstream chemistry.

Liquid-target technology also includes low-volume and less conventional systems that demonstrate the flexibility of this approach. Rh(NO_3_)_3_ solutions have been investigated for ^103^Pd production, [^66^Zn]Zn(NO_3_)_2_ for ^66^Ga, and an 80 µL tantalum target containing 10% [^13^C]methanol for [^13^N]ammonia production [55,111,112]. These systems are less central to current routine radiometal production, but they show that the term “liquid target” encompasses not only concentrated aqueous nitrate solutions but also specialized matrices and target chambers designed for relatively small volumes or specific nuclear reactions.

### 4.2. Radionuclidic-Impurity Pathways in Liquid Targets

Despite this technological diversity, the available studies reveal a limited number of recurring mechanisms by which radionuclidic impurities arise. The first originates from nuclear reactions within the target material itself. The actual proton-energy window extends from the incident energy to the exit energy from the solution, and therefore the desired production reaction is accompanied, depending on target isotopic composition and energy, by competing reaction channels. This mechanism produces ^66^Ga and ^67^Ga during ^68^Ga production, other Cu isotopes during Cu production, ^85^Y and ^85m^Y during ^86^Y production, ^58g^Co and ^56^Co during ^58m^Co production, and other Tc radionuclides in Mo-based systems. These impurities are particularly important because they are chemically identical to the desired product and cannot be removed by conventional chromatography or during radiolabelling. Their control must therefore precede chemical separation and relies principally on target isotopic composition, selection of the irradiation energy window, irradiation time, and, where useful, cooling time [5,8,36,37,70,71,72,73,74,75,76,110].

The second pathway is associated with the chemical composition of the target solution. A liquid-target matrix is not composed exclusively of the isotope participating in the desired nuclear reaction. Nitrate, solvent, acid, complex target compounds, additives, and other constituents introduce additional atoms into the beam field. Zn(NO_3_)_2_, Y(NO_3_)_3_, Sr(NO_3_)_2_, Fe(NO_3_)_3_, Ca(NO_3_)_2_, and phosphomolybdic acid contain N, O, P, and other elements that may themselves participate in nuclear reactions. Short-lived ^13^N and ^18^F have, for example, been observed in calcium systems. The chemical form of the target is therefore relevant not only to solubility and processing but also to the spectrum of species that may be generated during irradiation.

The same matrix composition determines the physicochemical processes that control whether a radionuclide formed within the target remains in solution, precipitates, adsorbs on an irradiated surface, or becomes associated with particulate or colloidal material. Water radiolysis produces H_2_, O_2_, and reactive species, while gas formation can reduce the effective proton path within the liquid and impair heat transfer. Irradiation-induced changes in local pH may promote hydrolysis and precipitation of metal hydroxides; the resulting precipitate may retain the radionuclide product or impurities, interfere with transfer, and cause nonuniform loading of the entrance window [2,74,81]. Consequently, solution stability must be established under actual irradiation conditions rather than inferred solely from pre-irradiation chemical stability.

Acidity provides a particularly clear example of the interaction between chemistry, target stability, and impurity control. In liquid ^68^Ga production, increasing HNO_3_ concentration improved reproducibility and yield at higher current, but a more acidic medium also imposes more stringent material-compatibility requirements on the target body, valves, tubing, cassette, and purification module. Nitrate solutions and niobium target bodies have therefore been selected in several ^86^Y and ^89^Zr systems to improve compatibility relative to less suitable matrices, while phosphomolybdic acid was shown to permit irradiation without visible degradation of the niobium target components under the investigated conditions [8,74,76,79,81,110]. The optimum target composition is thus not defined only by maximum radionuclide yield but by simultaneous control of radiolysis, pressure, precipitation, corrosion, radionuclide profile, and compatibility with subsequent purification.

A third potential source is activation of the entrance window, target body, and other materials surrounding the target solution. This pathway is very well characterized for the H_2_^18^O/^18^F system, in which irradiation of Havar, Nb, Ta, Ag, and other construction materials produces radionuclides that may subsequently be liberated into the target water. The same physical mechanism is applicable to other liquid targets because the proton beam and secondary radiation interact not only with the target solution but also with the target assembly. Products formed in these materials can potentially enter the liquid through nuclear recoil, sputtering, radiation-induced corrosion, thermal erosion, or particle detachment. However, the experimental characterization of such hardware-derived activation products is substantially less complete for emerging radiometal liquid targets than for routine ^18^F production. The absence of detailed activity measurements in individual process fractions should therefore not be interpreted as evidence that this pathway is absent.

The formation of a radionuclide within either the target matrix or the surrounding hardware does not itself determine whether it becomes an impurity of the precursor or final radiopharmaceutical. A further requirement is mobilization and transfer into the fraction directed to processing. In a liquid target, soluble species may be transferred directly with the bulk solution, whereas precipitated material, colloids, particles, material adsorbed on surfaces, or activity retained within the target assembly may follow different pathways. The target transfer therefore represents a partitioning step between activity remaining in the target system and activity entering the downstream module. This distinction is particularly important when radiolysis, corrosion, or precipitation changes the chemical or physical form of the activated species during irradiation.

### 4.3. Downstream Separation and Control of Radionuclidic Impurities

Once an impurity has entered the process stream, its fate is determined by its chemical form, matrix composition, sorbent selectivity, column capacity, acidity, washing conditions, and elution sequence. Liquid radiometal production generally requires separation of tracer-level activity from a macroscopic quantity of Zn, Ni, Ca, Sr, Y, Fe, Mo, or another target material. The purification system must therefore perform two related but distinct functions: recover the desired radionuclide efficiently and remove target material and radionuclides of other elements to a level compatible with radiolabelling. Multicolumn solid-phase extraction, ion-exchange, extraction chromatography, and matrix-exchange procedures can achieve substantial removal of chemically distinct radionuclides and stable metals. Their effectiveness does not, however, extend to isotopes of the desired element. ^66^Ga and ^67^Ga remain with ^68^Ga, other Cu isotopes remain with Cu products, ^85^Y and ^85m^Y remain with ^86^Y, Co isotopes remain with ^58m^Co, and other Tc radionuclides remain with ^94m^Tc.

This distinction has important implications for process design. Chemically separable activation products can, in principle, be redirected to columns, recovered target material, washing fractions, or waste by improving the downstream process. Chemically inseparable radionuclides must instead be prevented or minimized upstream. Maximum EOB activity is therefore not necessarily associated with maximum radionuclidic purity, and the irradiation conditions must be selected with the intended time of purification, radiolabelling, release, and administration in mind. For emerging theranostic systems, this requirement is particularly important because the impurity profile and residual elemental contamination may influence molar activity, radiolabelling performance, and consequently the comparability of the diagnostic member with the corresponding therapeutic radiopharmaceutical.

The liquid-target process must consequently be validated as an integrated sequence rather than as an irradiation step followed by independent purification. The relevant chain comprises target composition and isotopic enrichment, incident and exit energies, radiolysis and matrix stability, integrity of the entrance window and target body, transfer efficiency, chromatographic separation, recovery of expensive enriched material, matrix exchange, and functional radiolabelling. This explains why the current liquid-target approach is most mature for ^68^Ga, whereas applications to ^94m^Tc, ^64^Cu, ^44^Sc, ^86^Y, ^58m^Co, ^89^Zr and other radionuclides are more frequently used to extend the capabilities of standard medical cyclotrons where dedicated solid-target infrastructure is unavailable. The shared limitation is not simply the separation of a radiometal from the target matrix, but coordination of isotopic purity, solution stability, structural robustness, target-material recovery, and precursor quality.

### 4.4. Relationship of Liquid-Target Systems to the H_2_^18^O/^18^F Reference Model

The H_2_^18^O/^18^F system and the radiometal liquid targets described above differ substantially in target composition, solute concentration, acidity, product chemistry, purification strategy, and the relative importance of individual physicochemical processes. Nevertheless, the H_2_^18^O/^18^F system remains a useful reference because it demonstrates experimentally that the radionuclides generated during irradiation do not all follow the same pathway through the production process. Their relevance to the final product depends not only on whether they are formed, but also on whether they are released or remain retained within the irradiated system, whether they enter the fraction transferred to processing, and how they behave during subsequent separation and radiosynthesis. The value of the ^18^F model therefore lies in the sequence of processes that can be followed experimentally rather than in chemical similarity to other liquid-target systems.

Several direct analogies can be identified. Competing nuclear reactions occur within the irradiated liquid in both H_2_^18^O and radiometal targets, although the resulting radionuclide spectrum depends on the isotopic composition of the target material and the effective proton-energy range. In radiometal systems, Zn, Ni, Ca, Sr, Fe, Y, Mo, and other target elements may generate both the desired radionuclide and accompanying products through competing reaction channels. Additional atoms introduced by the chemical matrix may also participate in irradiation. Nitrate, acid, solvent, and more complex target compounds therefore influence not only solution stability but also the elemental inventory exposed to the beam. This is analogous to the formation of radionuclides from residual isotopes and other constituents of the H_2_^18^O target, although the greater chemical complexity of concentrated radiometal solutions increases the number of possible interactions.

A second common feature is the irradiation of the target surroundings. In the H_2_^18^O/^18^F system, activation of Havar, Nb, Ta, Ag, and other construction materials has been extensively characterized, together with the subsequent release of some activation products into the target water. The same physical processes may occur in radiometal liquid targets because the proton beam and secondary radiation interact not only with the target solution but also with the entrance window, target body, and other irradiated components. Radionuclides formed in these materials may enter the liquid through recoil, sputtering, radiation-induced corrosion, thermal erosion, or particle detachment. However, direct experimental characterization of these hardware-derived products remains substantially less complete for emerging radiometal liquid targets than for routine ^18^F production. The absence of detailed activity measurements in individual process fractions should therefore not be interpreted as evidence that this pathway is unimportant.

The principal difference lies in the physicochemical environment in which mobilization and transfer occur. In the H_2_^18^O/^18^F system, the irradiated medium contains only a relatively small elemental burden, whereas radiometal targets frequently contain concentrated metal salts in acidic solution. Radiolysis, gas evolution, local changes in pH and redox conditions, precipitation, adsorption, and colloid formation can consequently have a greater influence on the physical and chemical form of both the desired radionuclide and accompanying impurities. Soluble species may be transferred directly with the bulk solution, whereas precipitated material, colloids, particles, or radionuclides adsorbed on target surfaces may remain partly within the target system. The transfer from the irradiated target to the purification module therefore acts as a partitioning step between radionuclides that remain in the target assembly and those that enter downstream processing.

The subsequent chemical separation follows the same general principle observed in the ^18^F process: different radionuclides are retained, transmitted, or redirected according to their chemical form and the selectivity of the purification system. In H_2_^18^O/^18^F production, QMA and downstream purification components retain individual activation products to markedly different extents. In radiometal liquid-target production, ion exchange, solid-phase extraction, extraction chromatography, precipitation, and multicolumn procedures can similarly remove target material, stable metals, and radionuclides of chemically different elements. The greater target-solute concentration, however, increases the importance of sorbent capacity, breakthrough, matrix effects, co-elution, and recovery of enriched target material.

A fundamental limitation common to all of these systems is that chemical purification cannot separate isotopes or isomers of the same element. This limitation is comparatively more prominent in many radiometal liquid targets than in routine ^18^F production. Thus, ^66^Ga and ^67^Ga remain associated with ^68^Ga, other Cu radionuclides remain with Cu products, ^85^Y and ^85m^Y remain with ^86^Y, Co isotopes remain with ^58m^Co, and other Tc radionuclides remain with ^94m^Tc. Their contribution must therefore be minimized before downstream separation through control of isotopic enrichment, nuclear reaction, effective energy window, irradiation time, and, where appropriate, cooling time. In this respect, optimization of chemical purification cannot compensate for an unfavourable radionuclide composition established during irradiation.

Another important analogy concerns the final location of radioactivity. Neither the H_2_^18^O/^18^F system nor radiometal liquid targets can be evaluated solely on the basis of EOB activity or radionuclidic purity of the released product. Radionuclides removed from the product stream may remain in the target chamber, recovered target material, cartridges or columns, tubing, washing fractions, synthesis components, or liquid and solid waste. Consequently, successful purification represents redistribution of activity within the production system rather than its elimination. This distinction becomes particularly important when enriched target material is recovered and reused, because the recovered fraction may contain residual radioactivity or stable contaminants that can influence subsequent production cycles.

The H_2_^18^O/^18^F system is therefore useful as a reference not because other liquid targets reproduce its chemistry, but because it provides the most extensively characterized example in which radionuclide formation, release, transfer, separation, and redistribution can be followed through the complete production process. Emerging radiometal liquid targets modify the relative importance of these processes through differences in target composition, solution stability, isotopic enrichment, radiolysis, chromatographic loading, and recovery requirements. The same recurring processes are examined below for solid-target production, where post-EOB dissolution introduces an additional boundary between radionuclide formation and entry into the chemical process.

## 5. Radionuclides Produced in Solid Targets

### 5.1. Radionuclidic-Impurity Pathways in Solid Targets

As in liquid targets, the first source of radionuclidic impurities is the nuclear reaction occurring within the target material. The proton or other charged-particle beam traverses a finite target thickness and therefore experiences an energy interval rather than a single nominal energy. Desired and competing reaction channels may consequently operate simultaneously, and their relative contributions depend on excitation functions, incident and exit energies, target thickness, isotopic enrichment, beam profile, and irradiation time. The highest achievable yield does not necessarily correspond to the highest radionuclidic purity.

This limitation becomes particularly important when competing reactions generate isotopes or isomers of the same element as the desired product. In enriched ^68^Zn targets, higher energies and residual Zn isotopes contribute to ^67^Ga and ^66^Ga; residual isotopes in highly enriched ^100^Mo and the broad energy interval of thick targets generate other Tc isotopes and isomers; analogous problems occur for Cu, Sc, Y, Tb, Pb, La, Co, and Br. These radionuclides remain chemically indistinguishable from the desired product and therefore cannot be removed by conventional ion-exchange, extraction chromatography, or radiolabelling [5,6,7,8,9,10,17,18,19,20,21,22,23,24,25,26,27,28,29,30,31,35,44,45,46,54,56,57,58,61,62,63,64,113,114]. Consequently, isotopic enrichment, actual beam-energy window, target thickness, irradiation and cooling times, and the intended time of product use are primary upstream controls of radionuclidic purity.

The ^100^Mo(p,2n)^99m^Tc system provides a particularly clear demonstration of this principle. Thick highly enriched ^100^Mo targets permit high-current production, but reactions involving residual Mo isotopes and additional channels opened at higher proton energies produce other Tc radionuclides and isomers that accompany ^99m^Tc through chemical isolation. Increasing ^100^Mo enrichment and restricting the energy window are therefore more effective in controlling isotopic purity than downstream chromatography, which can remove Mo and radionuclides of other elements but cannot alter the isotope composition of technetium [9,10,113,114,115]. The same principle is observed for ^67^Cu produced from enriched ^70^Zn: Ga and Zn radionuclides can be removed chromatographically, whereas coproduced ^64^Cu remains with the product [17,69].

A second source of impurities is the activation of materials surrounding the active target layer. Solid-target assemblies may contain backing metals, encapsulation foils, adhesion layers, solder, seals, degraders, graphite, Mg, or other materials selected for thermal or mechanical reasons. Ag, Au, Al, Pt, Nb, In, graphite-associated materials, and Mg occur among the auxiliary materials described in solid-target systems. These components may undergo direct proton-induced reactions or secondary-neutron activation. If the active layer is thinner than intended, nonuniform, damaged, or misaligned with the beam, a greater proportion of the beam energy is deposited in the backing and the resulting activation profile changes. Importantly, activation products formed in these materials may remain physically immobilized during irradiation and become relevant to product quality only after the target is dissolved.

This behaviour creates a fundamental distinction between the formation and mobilization of solid-target activation products. A radionuclide formed in the backing or encapsulation is not automatically a process impurity. It becomes relevant to the crude product only when dissolution or another post-EOB operation releases it from its site of formation. The dissolution boundary is therefore a major impurity-control barrier. Selective dissolution of an electrodeposited or deposited active layer can leave much of the activated backing outside the process, whereas aggressive dissolution conditions or complete dissolution of a multilayer assembly may introduce radionuclides and stable elements from the backing, foil, seal, and auxiliary materials into the same solution as the desired product.

The composition of the active material itself may also influence impurity mobilization. Composite or chemically bound targets introduce elements that are not directly required for the desired nuclear reaction but affect target stability, thermal conductivity, or dissolution. Mg in 58Ni/Mg targets is an example: it improves target performance and facilitates dissolution by producing a porous Ni matrix, but it simultaneously increases the mass of material requiring downstream separation. Similarly, oxide, carbonate, chloride, and other chemical forms influence both the irradiated elemental inventory and the conditions required for target dissolution. Selection of the target compound is therefore an upstream decision with downstream consequences for both radionuclide and elemental purity.

Volatility constitutes a different mobilization mechanism. In Br production from solid Se-containing targets and in ^211^At production from Bi, the desired radionuclide may be recovered through volatilization and subsequent trapping rather than target dissolution. In such systems, containment, distillation, gas traps, and off-gas management are important barriers governing radionuclide transfer [32,33,57,58]. However, alternative chemical processing routes may also be applied, depending on the target composition and product properties.

The production of ^211^At illustrates the relationship between competing nuclear reactions and post-irradiation separation. ^211^At is produced through the ^209^Bi(α,2n)^211^At reaction, whereas higher α-particle energies may enable the competing ^209^Bi(α,3n)^210^At reaction. The resulting ^210^At cannot be separated chemically from ^211^At and decays to the long-lived α-emitter ^210^Po, making control of the effective irradiation energy essential. After irradiation, ^211^At can be recovered by dry distillation and cold trapping; in automated [^211^At]PSMA-5 production, neither ^210^At nor residual Bi was detected in the final product [32]. Alternatively, dissolution of irradiated Bi_2_O_3_ in 2 M HNO_3_ followed by selective precipitation of Bi(OH)_3_ provided approximately 80% ^211^At recovery with radionuclidic purity exceeding 99.9% [33]. These examples demonstrate that irradiation-energy control limits the formation of chemically inseparable isotopic impurities, whereas subsequent processing determines the separation of ^211^At from the target material and other chemically distinct contaminants.

An alternative cyclotron-based route for ^225^Ac production involves high-energy proton spallation of ^232^Th. Unlike direct production through the ^226^Ra(p,2n)^225^Ac reaction, this route generates a broad spectrum of radionuclides, including long-lived ^227^Ac. Because ^227^Ac is chemically inseparable from ^225^Ac, its presence represents a particular challenge for radionuclidic purity and cannot be effectively reduced by practical cooling periods. An alternative approach involves separating spallation-produced ^225^Ra and subsequently recovering ^225^Ac following radioactive decay. Thus, the two cyclotron-based routes illustrate how target composition and nuclear reaction pathways determine the radionuclidic impurity profile and the available strategies for its control [34].

### 5.2. Dissolution, Separation, and Final Distribution of Impurities

For most radiometal solid targets, dissolution is the decisive transition between the irradiated assembly and the chemical purification process. The acid composition and volume, temperature, dissolution time, order of reagent addition, and filtration of the crude solution determine which portions of the target assembly enter the downstream fraction. Selective dissolution is therefore not merely a convenient method of recovering the desired radionuclide; it functions as an additional impurity barrier that has no direct equivalent in a liquid target in which the irradiated medium itself is transferred from the target chamber.

After dissolution, the chemical problem resembles that encountered in liquid radiometal targets: tracer-level product activity must be isolated from a macroscopic target matrix together with radionuclides and stable elements originating from target material and auxiliary components. For ^64^Cu, ion-exchange procedures separate radiocopper from Ni, Co, and backing metals and permit recovery of costly enriched ^64^Ni. For Sc, DGA, Nobias, UTEVA, or cation-exchange media separate radioscandium from macroscopic Ca, V, or Ti. For ^86^Y, DGA extraction separates Y from Sr; for ^203^Pb, Pb-selective resin combined with Tl precipitation or secondary ion exchange separates the product from toxic Tl and backing-derived material; and for lanthanides, multistep extraction may be required to separate adjacent elements [18,19,20,21,22,23,24,25,26,27,28,29,30,31,44,45,46,47,48,49,50,116,117,118,119,120].

These procedures efficiently remove radionuclides of other elements when suitable chemical selectivity exists, but they do not correct an unfavourable radionuclide composition established during irradiation. Other Sc isotopes remain with ^43^Sc, ^44^Sc, or ^47^Sc; other Tb isotopes, including ^153^Tb and long-lived ^157^Tb, remain with ^155^Tb; ^201^Pb remains chemically associated with ^203^Pb; La isotopes remain together after Ba removal; and Br isotopes remain together after thermochromatographic isolation [28,29,30,31,44,45,46,54,57,58,61,62,63]. The resulting radionuclidic purity is consequently determined jointly by upstream reaction control and radioactive decay rather than by chemical separation alone.

Target-material recovery is an additional characteristic of solid-target production because many routes employ highly enriched and costly materials. Recovery of ^68^Zn, ^64^Ni, Ti, Tl, Cr, Sn, and other materials is therefore part of the manufacturing cycle rather than solely an economic consideration [6,17,28,29,30,31,38,39,40,51,52,53,116,117,118,119,120]. Recycled material must retain suitable isotopic and chemical composition because accumulation of stable contaminants or changes in isotopic composition may affect subsequent radionuclide production and radiolabelling. The recovery fraction also constitutes a potential reservoir of residual radioactivity and must be included when evaluating the distribution of activity after processing.

Solid targets further demonstrate that purification may depend on time as well as chemical selectivity. Cooling allows short-lived impurities to decay but does not eliminate radionuclides with similar or longer half-lives than the desired product. In parent–daughter routes, delayed separation may instead be deliberately used to permit daughter in-growth. The ^225^Ac/^226^Ac system is an example in which a two- to three-week cooling interval and secondary separation contribute directly to final radionuclidic purity [34]. The process specification must therefore define not only irradiation and chemical-separation parameters but also the permitted interval between EOB, target processing, purification, radiolabelling, release, and administration.

The distribution of radioactivity after successful purification should also be considered explicitly. High radionuclidic purity of the final precursor can coexist with substantial activity retained in the backing, undissolved target material, recovered enriched material, dissolution vessel, filters, chromatographic resins, cassettes, and liquid or solid waste. In direct ^99m^Tc production, for example, numerous radionuclides were identified in the waste even though the isolated product met high radionuclidic-purity specifications [9,10,121]. Chemical purification therefore redistributes activity rather than eliminating it from the production system.

### 5.3. Relationship of Solid-Target Systems to the H_2_^18^O/^18^F Reference Model

The same four-stage framework applies to solid-target production, although the timing and mechanism of Stage II differ fundamentally from those in the H2^18^O/^18^F system. In liquid targets, mobilization and transfer may occur during irradiation or target emptying, whereas in solid targets a substantial fraction of the radionuclide inventory remains physically immobilized until post-EOB dissolution or thermal processing.

The principal difference lies between formation during irradiation and entry into the process stream after irradiation. In H_2_^18^O/^18^F production, radionuclides formed in the liquid or released from the window and target body may already be present in the transferred target water at EOB. Radiolysis, corrosion, recoil, sputtering, and particle detachment can therefore influence impurity transfer during irradiation. In a solid target, by contrast, a substantial proportion of the target material and its activation products remains physically immobilized until post-EOB dissolution. The dissolution step creates an additional boundary at which activated backing, encapsulation, seal, degrader, or auxiliary materials can either remain outside the product stream or be mobilized together with the desired radionuclide.

There are nevertheless important common features. In both systems, isotopic composition and the actual energy window determine which radionuclides are formed; auxiliary target materials may become activated; product quality depends on which activated species are mobilized rather than on the complete inventory of radionuclides formed in the installation; and the final fate of mobilized impurities is governed by the selectivity and capacity of subsequent purification barriers. In both cases, isotopes of the same element as the desired product represent the most difficult category because conventional chemical purification cannot separate them. Their control must therefore occur upstream through selection of the reaction, isotopic enrichment, energy window, target thickness or concentration, irradiation and cooling times, and the allowable time to use the product.

Solid targets add two features that are less prominent in the H_2_^18^O model. The first is the dissolution boundary, which may act as an additional separation barrier if the active material can be selectively removed while activated construction materials remain intact. The second is the substantially larger elemental burden associated with the target matrix and backing. Once mobilized, stable Zn, Ni, Ca, Sr, Y, Gd, Tl, Ba, Fe, Cu, Al, or other metals can increase the load on separation media and may compete with the desired radiometal during subsequent chelation even when they have no direct radionuclidic significance [122].

### 5.4. Comparison of Radionuclidic Impurity Pathways in Liquid and Solid Targets

The preceding sections examined radionuclidic impurity pathways in liquid- and solid-target production separately, using the H_2_^18^O/^18^F system as a reference model. Table 3 provides a direct comparison of these systems, highlighting both the mechanisms common to different target types and the differences arising from target composition, impurity mobilization, and subsequent processing. Representative purification procedures and reported experimental results are included to illustrate the practical implications of these differences.

Table 3 summarizes the similarities and differences in radionuclidic impurity formation, mobilization, transfer, and control between the H_2_^18^O/^18^F reference system, other liquid targets, and solid targets. Representative radionuclides and purification procedures are included to illustrate how the common mechanisms are influenced by target composition, physicochemical conditions, and downstream processing. The comparison highlights the applicability of the ^18^F-based mechanistic framework to different cyclotron-production technologies while identifying the target-specific factors that determine impurity behaviour and the effectiveness of their control.

The co-production of isotopes of the same element as the desired radionuclide represents a particular challenge because they cannot be removed by conventional chemical purification. The examples of ^68^Ga, ^44^Sc, and ^86^Y illustrate how target composition, proton-energy selection, and radioactive decay influence radionuclidic purity.

In liquid-target production of ^68^Ga, a 1.0 M solution of enriched [^68^Zn]Zn(NO_3_)_2_ in 0.2–0.3 M HNO_3_ was irradiated with protons at a nominal energy of 14.3 MeV [76]. Although the competing ^68^Zn(p,2n)^67^Ga reaction becomes energetically possible above approximately 12.16 MeV, the selected energy limited ^67^Ga production while maintaining an acceptable ^68^Ga yield. Additional ^66^Ga and ^67^Ga may originate from residual Zn isotopes. Radionuclidic purity typically exceeded 99.8% at EOB, while longer-lived Ga impurities were assessed by gamma-ray spectrometry after 24–72 h of decay. Because both impurities have longer half-lives than ^68^Ga, delaying product use increases their relative contribution.

For solid-target production of ^44^Sc, two irradiation conditions using enriched ^44^CaO were compared, with proton energies of approximately 10.3 and 13 MeV [19]. Gamma-ray spectrometry showed approximately 0.5% and 2% of the co-produced ^44^mSc, respectively. Both energies were below the approximately 14.46 MeV threshold for the ^44^Ca(p,2n)^43^Sc reaction, but ^44^mSc was produced through the same (p,n) channel as ^44^gSc. Trace amounts of ^88^Y, attributed to Sr impurities in the target material, were also identified. This comparison illustrates that energy selection can influence the production of isomeric impurities without eliminating them completely.

For ^86^Y, production from enriched ^86^SrCO_3_ was investigated using a 14.6 MeV medical cyclotron [26], while another approach employed ^86^SrCl_2_ and proton-energy degraders to obtain the required lower irradiation energies [27]. Gamma-ray spectrometry was used to evaluate production yields and accompanying Y radionuclides. The competing ^86^Sr(p,2n)^85^Y reaction has a threshold of approximately 15.7 MeV and can therefore be limited by selecting an appropriate energy window. Unlike the longer-lived Ga impurities accompanying ^68^Ga, the shorter-lived ^85^gY and ^85^mY may also be reduced relative to ^86^Y by allowing sufficient decay time, although this results in a simultaneous loss of product activity.

The representative examples presented in Table 3 also illustrate the diversity of purification approaches investigated for cyclotron-produced radionuclides. For liquid-target production of ^68^Ga, a two-column solid-phase extraction procedure yielded more than 50% of the isolated [^68^Ga]GaCl_3_ [76], while subsequent developments investigated modified ZR resin configurations and elution with 1.75 M HCl [77]. For ^61^Cu produced from liquid Zn targets, automated purification using two extraction-chromatography resins and a strong anion exchanger provided an average yield of 63 ± 15%, with residual Zn assessed by ICP-MS [37]. Solid-target processing introduces additional separation requirements. For example, tandem anion-exchange and hydroxamate columns enabled ^89^Zr recovery of 95.1 ± 1.3% from irradiated Y targets [13], whereas a DGA/Dowex 50Wx8 procedure for ^44^Sc achieved a radiochemical yield of 95 ± 3% [18].

These studies demonstrate the continued development and optimization of radionuclide-specific separation procedures, including sorbent selection, chemical conditions, multicolumn systems, and automation. However, the reported performance indicators are not directly comparable, as they refer to different target matrices, processing conditions, and definitions of recovery or purity, and do not necessarily quantify the removal of individual radionuclidic impurities. A comprehensive comparison of separation chemistry and performance across individual production routes would therefore represent a distinct, more narrowly focused review. In the present mechanistic synthesis, these examples primarily illustrate how purification controls the transfer and distribution of chemically separable radionuclides, while coproduced isotopes of the product element remain dependent on upstream control.

## 6. Mechanisms of Impurity Formation and Introduction into the Process: A Stage-Based Framework

**Figure 3:**
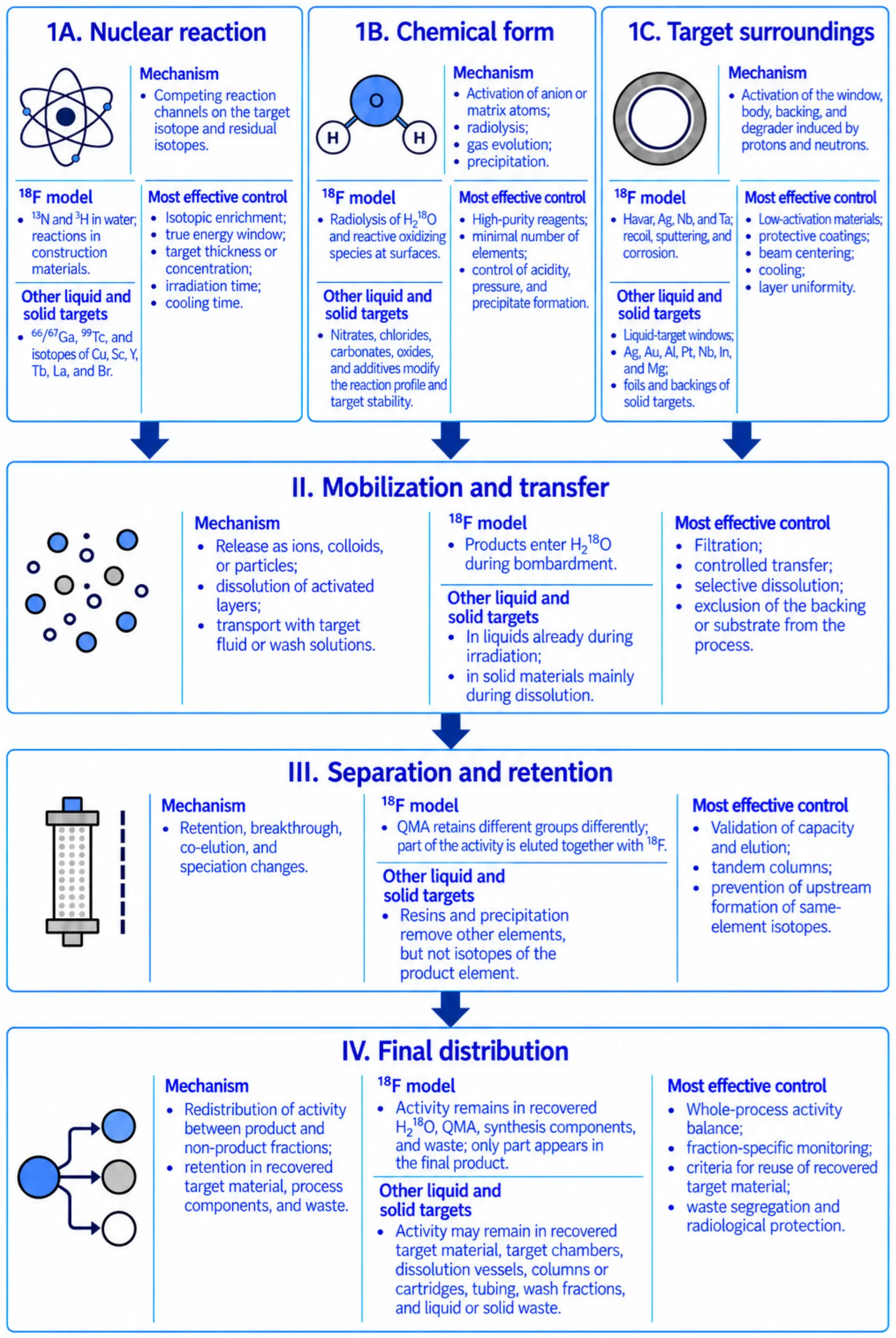
Stage-based model of impurity formation, transfer, and prevention.

The preceding sections show that the presence of a radionuclide within an irradiated target assembly does not necessarily imply contamination of the radionuclide precursor or finished radiopharmaceutical. Impurity pathways can be described by four consecutive stages: formation of by-product radionuclides, their mobilization and transfer into the crude post-irradiation fraction, separation and retention during purification and radiosynthesis, and final distribution between product and non-product fractions. (Figure 3) This framework applies to both liquid- and solid-target production routes, although the mechanisms operating within individual stages are radionuclide- and target-specific [1,2,3,4,83,84,85,86,87].

Stage I: Formation of By-Product Radionuclides

This stage defines the radionuclide inventory generated during irradiation. By-products may originate from competing reactions involving the intended target isotope or residual isotopes, reactions with other elements present in the chemical target matrix, and activation of target-surrounding materials such as windows, target bodies, backings, encapsulation layers, degraders, or other auxiliary components. The resulting inventory is determined primarily by the isotopic and elemental composition of the irradiated system and by the effective irradiation conditions. Importantly, formation alone does not determine whether a radionuclide will subsequently contaminate the process stream [1,2,3,4,83,84,85,86,87,113,114,115,116,117,118,119,120,125].

Stage II: Mobilization and Transfer into the Crude Post-Irradiation Fraction

A radionuclide formed during irradiation becomes relevant to downstream product quality only if it is mobilized from its site of formation and enters the fraction subjected to chemical processing. In liquid targets, this transfer may occur during irradiation or target emptying, whereas in solid targets it is commonly associated with post-EOB dissolution or thermal processing. Stage II therefore represents the boundary between the total radionuclide inventory generated within the target system and the subset of radionuclides that actually enters the crude product fraction [29,41,49,72,83,84,85,86,87,103,104,116,117,118,119,120].

Stage III: Separation and Retention during Purification and Radiosynthesis

Once transferred into the crude fraction, impurities are redistributed according to their chemical form and the selectivity of the applied separation process. Radionuclides of elements different from the desired product can often be removed by ion exchange, extraction chromatography, precipitation, distillation, or related methods. In contrast, coproduced isotopes and isomers of the product element generally follow the desired radionuclide through conventional chemical purification and therefore require primarily upstream control. Stage III determines which components of the transferred radionuclide inventory remain associated with the radionuclide precursor or proceed into radiosynthesis [5,6,7,8,9,10,17,18,19,20,21,22,23,24,25,26,27,28,29,30,31,44,45,46,57,58,69,70,71,72,73,74,75,76,77,78,79,80,81,82,83,113,114,115,116,117,118,119,120].

Stage IV: Final Distribution between Product and Non-Product Fractions

The final stage describes the location of radioactivity after processing. Activity may be present in the released radiopharmaceutical, recovered target material, purification media, synthesis components, filters, cassettes, vessels, or liquid and solid waste. Consequently, successful removal of an impurity from the product does not eliminate it from the manufacturing system but transfers it to another fraction. The final assessment should therefore distinguish radionuclidic purity of the product from the overall distribution of radionuclides generated during production and their implications for material recovery, radiation protection, and radioactive-waste management [9,10,83,88,89,97,121].

Together, these four stages provide a common framework for interpreting impurity pathways across cyclotron-production technologies: formation determines what is generated, transfer determines what enters the process, purification determines what accompanies the product, and final distribution determines where the remaining activity resides.

## 7. Analytical Characterization and Validation of Impurity Control

Analytical characterization should verify the impurity pathways defined in the stage-based framework rather than merely confirm the purity of the final product. Because radionuclides and elemental contaminants differ in radiation type, half-life, chemical form, and distribution within the production system, complementary radiometric, elemental, and functional methods are required. During process development, measurements should distinguish impurities generated during irradiation from those transferred into the crude fraction, retained during purification, or redirected to non-product fractions.

Gamma-emitting radionuclides are most commonly identified and quantified by high-purity germanium (HPGe) gamma-ray spectrometry. Measurements should use a defined geometry and a specified reference time relative to EOB, with decay correction when different fractions or production conditions are compared. Short-lived radionuclides require prompt analysis, whereas delayed measurements improve the detection of longer-lived components. Gamma spectrometry must be supplemented by radionuclide-specific techniques when photon emissions are absent or insufficient; for example, pure beta emitters such as tritium require liquid scintillation counting. The analytical method, sampling time, and detection limit should therefore be selected according to the expected impurity inventory [69,84,85,86,89,92,93,94,95,96,97,101,106].

Elemental impurities should be assessed using ICP-MS, ICP-OES, or other matrix-appropriate methods. For radionuclide precursors intended for radiolabelling, elemental concentrations alone are insufficient because trace metals below toxicological limits may still compete with the desired radionuclide at tracer-scale chelator concentrations. Elemental analysis should therefore be complemented, where appropriate, by functional radiolabelling tests assessing radiolabelling yield, apparent or effective molar activity, and complex stability [6,7,8,17,28,29,30,31,46,65,67,69,88,116].

Evaluated nuclear data and particle- or ion-transport modelling can support prediction of the expected radionuclide inventory and help define analytes, sampling points, and measurement times, but they do not replace experimental characterization. During process development and validation, analysis should extend beyond the final product to representative process fractions required to confirm impurity transfer and retention. Routine batch release, in contrast, should focus on attributes directly related to product specifications. Thus, analytical characterization serves two distinct purposes: confirmation of radiopharmaceutical quality and validation of impurity-control mechanisms throughout the production process [25,26,27,35,80,84,103,105,120,126,127,128,129,130,131,132,133].

## 8. Strategy for the Prevention and Control of Radionuclide Impurities

Impurity control should begin before the first irradiation. The preferred hierarchy comprises prevention of formation, limitation of release and mobilization, selective isolation of the radionuclide precursor after EOB, and use of radiosynthesis and final purification to remove chemical and radiochemical impurities. Interventions applied at late stages cannot compensate for an inappropriate nuclear reaction or isotopic composition and may increase processing time, product losses, occupational exposure, and waste generation [1,2,3,4].

Impurity control should follow the production pathway defined in the preceding stage-based framework, with priority given to preventing impurity formation and transfer rather than relying on downstream removal. Upstream control therefore includes selection of the nuclear reaction, target isotopic and chemical composition, effective energy window, irradiation and cooling conditions, and materials of the complete target assembly. These parameters should be defined and validated before routine production, because radionuclides generated from competing reactions or from activated target materials cannot always be removed at later processing stages [1,2,3,4,5,6,7,8,9,10,17,18,19,20,21,22,23,24,25,26,27,28,29,30,31,35,44,45,46,50,54,56,57,58,60,61,62,63,64,72,76,80,86,87,90,91,114,115,116,117,118,119,120,121,122,123,124,125,126,127,128].

Three general impurity-control situations should then be distinguished:

- First, radionuclides and stable elements belonging to an element other than the product are often chemically separable. The activation products of ^18^F production and residues of Zn, Ni, Fe, Co, Ca, Sr, Y, Gd, Tl, or Ba in radiometal production can be reduced by filtration, precipitation, ion exchange, extraction chromatography, distillation, or tandem-column purification. The limiting factors include chemical speciation, sorbent capacity, breakthrough, coelution, matrix composition, and product recovery [5,6,7,8,11,12,13,14,15,16,17,28,29,30,31,44,45,46,65,67,69,70,71,73,74,75,76,77,78,79,80,81,82,83,88,117,118,120].
- Second, the contribution of selected short-lived products can be reduced by a validated cooling period. This approach is useful for short-lived radionuclides formed in water or calcium-based systems and, on a different timescale, for routes in which decay and a second separation step reduce the contribution of a longer-lived parent or coproduced radionuclide. Cooling is effective only when the impurity decays substantially faster than the product does, and the delay remains compatible with the required activity and clinical schedule [19,34,72,95,96,97,98].
- Third, the isotopes and isomers of the product element are chemically inseparable via conventional radiochemical methods. Examples include ^66,67^Ga accompanying ^68^Ga, other Tc isotopes accompanying ^99m^Tc, Cu isotopes accompanying ^64^Cu or ^67^Cu, Sc isotopes accompanying ^43,44,47^Sc, ^85^Y accompanying ^86^Y, other Tb isotopes accompanying ^155^Tb, ^135^La accompanying ^132,133^La, and Br isotopes accompanying ^76,77,80m^Br. Their control is primarily physical and nuclear and includes isotopic enrichment, selection of the reaction and energy window, target thickness, irradiation and cooling times, and limitation of product shelf life [5,6,7,8,9,10,17,22,23,24,25,26,27,35,44,45,46,54,57,58,61,62,63,64,114,115,116,121].

Post-EOB purification should remove the bulk target matrix and chemically separable impurities while providing the radionuclide precursor in a form suitable for radiolabelling. Process performance should be evaluated in terms of product recovery, radionuclidic and elemental purity, matrix compatibility, and representative radiolabelling performance.

Subsequent radiosynthesis and final purification can remove chemically distinct radionuclides, free radionuclide species, radiochemical byproducts, and residual target-derived contaminants, but they cannot correct the isotopic composition established during irradiation. Product-release criteria should therefore remain linked to the validated upstream production conditions and the permitted shelf life. In addition, rejected activity should be accounted for in process components and waste streams as part of the overall impurity-control strategy [9,17,65,67,69,83,88,89,116,117,118,120,121].

## 9. Implications for Radiation Protection and Radioactive Waste Management

Radionuclides excluded from the released product remain within the production system and may therefore contribute to occupational exposure, radioactive waste, and long-term activation of equipment. Radiation protection and waste management should consequently be considered as integral components of radionuclide-production and purification design [83,85,89,93,121,133,134,135,136,137,138,139,140,141,142,143,144].

### 9.1. Redistribution of Activity and Occupational Exposure

Occupational exposure is particularly relevant during post-irradiation target handling, emptying or dissolution, connection and disconnection of process lines, removal of columns and cassettes, management of incomplete transfers, and servicing of activated components. Remote handling, shielding, automation, and delayed intervention reduce exposure, but activity may remain concentrated in small process components or hidden volumes. Radiation-protection assessment should therefore consider the actual task geometry and the distribution of activity within the process rather than relying solely on the activity of the final product [3,10,24,28,29,30,31,36,37,72,76,77,78,79,107,114,115,116,117,118,120,121].

The ^18^F process illustrates this distribution particularly clearly. Activation products may partition between recovered H_2_^18^O, the QMA cartridge, downstream purification media, the reactor, and liquid waste. ^51^Cr and ^7^Be are retained to a large extent on QMA, part of the Co, Mn, and Ni activity passes into recovered water, whereas Nb, Ta, Re, and Tc species may be retained and subsequently partly eluted into the reaction vessel. Thus, the finished radiopharmaceutical may satisfy radionuclidic-purity requirements while cassettes, sorbents, recovered water, and waste remain radioactive [83,85,88,89,92,93,94,95,96,97,98,99,100,106,107,108].

### 9.2. Long-Lived Activation Products and Waste Classification

Radioactive waste generated during cyclotron production should be classified according to its complete radionuclide inventory, activity concentration, half-lives, and requirements for containment and disposal, rather than the half-life of the desired product alone. The IAEA classification framework (General Safety Guide No. GSG-1) distinguishes exempt waste (EW), very short lived waste (VSLW), very low level waste (VLLW), low level waste (LLW), intermediate level waste (ILW), and high level waste (HLW). For medical cyclotron production, VSLW, VLLW, and LLW are particularly relevant, although assignment to a specific class requires radionuclide-specific characterization and compliance with national regulatory criteria. VSLW may be managed by storage for decay followed by authorized clearance, whereas waste containing persistent activation products may require longer-term controlled storage or disposal through an appropriate radioactive-waste management route.

Segregation should be performed at the point of generation according to radionuclide composition, activity, half-life, physical and chemical form, and non-radiological hazards, consistent with IAEA Specific Safety Guide No. SSG-45. Typical streams include aqueous target residues and recovered water, organic solvents, used ion-exchange and extraction-chromatography media, filters, synthesis cassettes, and activated target components. These streams should not automatically be assigned to the same waste category. For example, cartridges containing predominantly short-lived activity may be suitable for decay storage, whereas sorbents, target residues, or activated metal components containing longer-lived radionuclides may require separate management. Potential activation products include ^3^H, ^54^Mn, ^57^,^58^,^60^Co, ^22^Na, ^65^Zn, ^110m^Ag, ^207^Bi, and ^26^Al. Their radiological significance differs: tritium is primarily relevant to internal contamination and water management, while radionuclides such as ^60^Co and ^22^Na may remain sources of external exposure after the desired product has decayed [95,96,97,121,136,138,139,140,141,142,143].

Decay storage is appropriate when the radionuclide inventory and its evolution over time demonstrate that the material can meet the applicable clearance criteria within a practicable storage period. The required period depends on the half-lives and initial activities of all relevant radionuclides, including long-lived impurities and radioactive progeny where applicable; it cannot be determined from the desired radionuclide alone. Containers should remain identified and appropriately shielded or contained during storage, and activity records, decay calculations, and suitable measurements should support the decision on subsequent management. In the European Union, clearance from regulatory control is governed by Article 30 and Annex VII of Council Directive 2013/59/Euratom, as implemented in national legislation. Storage for a predetermined number of half-lives does not, by itself, establish eligibility for clearance.

The H_2_^18^O/^18^F system illustrates these considerations. Although most ^18^F activity decays rapidly, recovered target water and purification residues may contain longer-lived activation products requiring separate characterization. Distillation can remove many gamma-emitting and chemical contaminants from recovered H_2_^18^O but does not selectively remove tritiated water (HTO), which can accompany the recovered water fraction. Similarly, purification cartridges and other process components may retain radionuclides that are absent from the released radiopharmaceutical. Waste classification and management should therefore be based on the measured or otherwise adequately established radionuclide composition of each stream, with recovered target material evaluated separately before reuse [83,85,88,89,98,99,100,121].

### 9.3. Activated Facility Components, Monitoring, and Activity Balance

Activation is not restricted to the material transferred into the chemical process. Radionuclides may remain in target windows and bodies, degraders, collimators, deflectors, coils, yokes, cooling circuits, and shielding. Secondary neutrons generated during target irradiation and competing nuclear reactions may also activate materials outside the direct beam path. Because such activation may persist for years or decades, it is relevant to routine maintenance, component replacement, and eventual decommissioning [133,134,135,136,137,138,139,140,141,142,145,146,147].

Monitoring should combine dose-rate and surface-contamination measurements with radionuclide-specific methods such as gamma-ray spectrometry and, where required, liquid scintillation counting. During process development, an activity balance should account for the irradiated target, transferred fraction, product, recovered material, purification media, filters, traps, and waste. Failure to close the balance may indicate incomplete transfer, sorbent breakthrough, retention in tubing, unrecognized contamination, or accumulation in reusable components [85,89,97,121,133,134,135,136,137,138,139,140,141,142,143,144].

For emerging production routes, the waste-management strategy, activity balance, and task-specific radiation-protection assessment should be established before progression to higher beam currents or routine clinical production.

## 10. Integrated Assessment of the Production Process and Lifecycle Oversight

The evidence reviewed here supports a five-domain framework for process assessment and validation that complements, rather than replaces, the mechanistic model described in Section 6:

- Nuclear reaction analysis: identification of the primary production route, competing reactions, threshold energies, relevant radionuclidic impurities, and the effective energy range within the target.
- Target and activation analysis: evaluation of the target medium and irradiated construction materials, including windows, target bodies, backings, degraders, and other auxiliary components that may contribute activation products.
- Mobilization and separation analysis: assessment of radionuclide release, transfer into the process stream, retention on purification media, transfer to the product, and distribution between recovered material and waste.
- Analytical and functional validation: integration of radionuclidic and elemental analyses, decay measurements, and representative radiolabelling tests with nuclear-reaction and particle-transport predictions.
- Whole-process assessment: evaluation of product quality, activity recovery, waste generation, occupational exposure, accumulation in reusable components, target-material recycling, and long-term activation of facility components.

Unlike end-product quality control, this framework considers impurity generation and activity distribution across the entire manufacturing process, including non-product fractions and retained activity. Product compliance therefore represents only one component of process control and should be interpreted together with information on impurity transfer, removal, and accumulation [1,2,3,4,83,84,85,86,87,89,97,121,133,134,135,136,137,138,139,140,141,142,143,144].

Assessment of a production route for radiopharmaceutical implementation should consider not only EOB yield but also achievable radionuclidic, radiochemical, and chemical purity, molar activity, radiolabelling performance, occupational exposure, and characterization of relevant waste streams. Demonstration of the nuclear reaction or a high EOB yield alone is insufficient to establish the suitability of the complete manufacturing process.

Validation should address relevant worst-case operating conditions, which may include minimum target enrichment, maximum beam current or irradiation time, maximum target loading, recycled target material, aged target components, high purification-column loading, and the longest acceptable interval before radiolabelling or product release. Changes in target composition, target hardware, process materials, purification media, automation parameters, or beam conditions should trigger assessment of their potential impact on impurity generation, distribution, product quality, occupational exposure, and waste management. Revalidation is warranted when such changes may alter the radionuclide or elemental impurity profile, even if product yield remains unchanged.

## 11. Conclusions

Production of ^18^F from H_2_^18^O provides the most extensively characterized model for understanding radionuclidic-impurity pathways in cyclotron production. Decades of routine use have shown that the radionuclide inventory generated during irradiation cannot be attributed solely to the intended nuclear reaction. By-product radionuclides may arise from competing reactions in the target isotope and residual isotopes, from reactions involving other atoms present in the target material or chemical matrix, and from activation of the entrance window, target body, and other construction materials. The ^18^F system further demonstrates that formation alone does not determine contamination of the final radiopharmaceutical: activation products must become mobile, enter the fraction directed to processing, and subsequently survive the implemented separation and purification steps. Their removal from the product stream results in redistribution to recovered target material, purification components, or waste rather than disappearance from the production system.

This mechanistic structure provides a general framework for evaluating emerging cyclotron-production routes. For any new radionuclide, impurity assessment should therefore begin by identifying the radionuclides that may be generated from the target isotope, the complete chemical matrix, and the irradiated target assembly, followed by evaluation of their mobilization, transfer into the crude post-irradiation fraction, behaviour during chemical separation and radiosynthesis, and final distribution between product and non-product fractions. The relative importance of these processes differs between liquid and solid targets: in liquid targets, impurity transfer may begin during irradiation, whereas in solid targets a substantial fraction of the radionuclide inventory may remain immobilized until dissolution or thermal processing.

The most difficult impurities remain isotopes and isomers of the product element because conventional chemical purification cannot separate them from the desired radionuclide. Their contribution must therefore be minimized primarily through upstream control of the nuclear reaction, target isotopic composition, effective energy window, target thickness or concentration, irradiation time, and, where applicable, cooling time. Radionuclides of other elements can often be reduced by selective chemical methods, although their removal redistributes activity within the manufacturing system and may contribute to occupational exposure and radioactive-waste generation.

The practical implication of this framework is that development of a new cyclotron-production route should not focus solely on EOB yield or on the radionuclidic purity of the final vial. Production planning should consider the complete sequence from nuclear reaction and target design through impurity mobilization, transfer, purification, analytical characterization, activity recovery, radiation protection, and waste management. The H_2_^18^O/^18^F system is therefore valuable not because other radionuclides reproduce its chemistry, but because routine experience with this target has demonstrated experimentally that all of these stages can determine whether an activation product becomes a product impurity or remains elsewhere in the production system.

For theranostic radionuclides, both members of the pair should additionally be compared in terms of radionuclidic and chemical purity, molar activity, radiolabelling yield, complex stability, availability for administration, and the radiation dose associated with impurities. The appropriate criterion of technological maturity is not the activity obtained at EOB, but a reproducible, radiolabelling-compatible, safe, and fully characterized manufacturing chain extending from the target material to the radiopharmaceutical and waste.

An important finding of this review is the presence of persistent evidence gaps that limit comparison of production technologies. These include incomplete reporting of the actual energy window within the target, target and construction materials, analytical methodology and detection limits, impurity-removal performance, and radiolabelling outcomes. Radiation-protection data are particularly scarce for emerging radionuclide-production routes, especially with respect to task-specific occupational exposure during target handling and processing, long-term activation of equipment and facilities, and activity accumulation in reusable process components and waste streams. Harmonized reporting standards would substantially improve reproducibility, facilitate comparisons between production technologies, and strengthen the translational assessment of emerging radionuclide-production methods.

## Figures and Tables

**Table 2:** Bibliographic search strategies.

Strategy	Thematic Scope	Main Search Terms
1	Therapeutic and theranostic radionclides	therap*; radionuclide; cyclotron
2	Liquid targets	“liquid target”; “solution target”; cyclotron
3	Impurities associated with ^18^F	impurit*; “activation product*”; F-18; 18F
4	Impurities and activation products in cyclotron production	impurit*; radionuclid*; “activation product*”; radioisotop*; cyclotron

**Table 3:** Similarities and differences in the mechanisms of radionuclidic impurity formation, transfer, and control relative to ^18^F production from H_2_^18^O.

Aspect Considered	Reference Model: ^18^F from H_2_^18^O	Other Liquid Targets	Solid Targets
Formation of by-product radionuclides	In addition to ^18^F, ^13^N is formed from residual ^16^O, together with activation products from the Havar entrance window and target body (e.g., ^51^Cr, ^52^Mn and Co radionuclides).	Similar competing nuclear reactions occur, but coproduction of isotopes of the product element is particularly important, e.g., ^66^Ga and ^67^Ga during ^68^Ga production or other Cu isotopes during ^61^Cu production.	Similar reactions occur in the target material, e.g., formation of other Tc isotopes during ^99m^Tc production from ^100^Mo. Backings and encapsulation materials may provide additional sources of activation products [123].
Influence of target composition and physical form	^18^O-enriched water is a relatively simple matrix. The mobility of activation products depends, among other factors, on radiolysis and interactions with construction-material surfaces.	Concentrated salt and acid solutions provide a more complex chemical environment. For example, ^68^Ga has been produced from [^68^Zn]Zn(NO_3_)_2_ solutions in HNO_3_; acidity and Zn concentration influence solution stability and irradiation conditions [8,76].	The chemical composition and physical form of the target (e.g., ^64^Ni electrodeposited on Au, ^100^Mo, or CaCO_3_) affect both activation of the materials and subsequent dissolution [124].
Impurity mobilization and transfer	Activation products released from the Havar window or target body may enter the water during irradiation and subsequently be transferred to the synthesis module.	A similar transfer mechanism occurs with the target solution, although some radionuclides may remain in precipitates, colloids, or adsorbed on target surfaces.	A key difference is that activation products may remain associated with the irradiated target assembly. Subsequent target dissolution or thermal processing determines whether they enter the downstream process.
Role of purification operations—examples	QMA Light retains [^18^F]fluoride and more than 90% of ^51^Cr and ^7^Be present in the irradiated water. During fluoride elution, some radionuclides remain retained, whereas Nb, Ta, Re and Tc radioisotopes may co-elute with [^18^F]fluoride [83]. Further purification depends on the radiopharmaceutical and its production process.	^68^Ga: a hydroxamate resin [8] and a two-column solid-phase extraction (SPE) system (ZR and TK200 resins) [76] have been used. ^61^Cu: a three-column system (two extraction chromatography resins and a strong anion exchanger) separated Cu from the Zn matrix [37]. ^103^Pd: anion-exchange chromatography separated Pd from Rh [55]. The reported activity yield of isolated [^68^Ga]GaCl_3_ was typically >50% for the two-column procedure [76], while the mean ^61^Cu purification yield was 63 ± 15% [37]. These methods do not remove coproduced isotopes of the product element.	^64^Cu: AG MP-50 cation-exchange resin with HCl/acetone mixtures separated Cu from Ni and radiocobalt [120]. ^44^Sc: DGA and Dowex 50Wx8 resins separated Sc from Ca and other metals [18]. Selective dissolution may additionally limit the release of backing-derived activation products. A decay-corrected radiochemical yield of 95 ± 3% was reported for the ^44^Sc separation procedure [18]. Chromatography can remove radionuclides of other elements, but not coproduced isotopes of the product element.
Final distribution of activity	By-product radionuclides may remain in recovered H_2_^18^O, the QMA cartridge, synthesis module and waste streams.	Activity may remain in recovered target-material solution, chromatographic columns and other post-processing fractions.	In addition to chromatographic columns and waste, a substantial fraction of the activity may remain in undissolved backing material or other target components.

## Data Availability

The data supporting the findings of this review are derived from publicly available publications cited in the article. The complete dataset generated during the literature search and screening process, including records subsequently excluded during study selection, is provided in the Supplementary Materials.

## Supplementary Materials

The following supporting information can be downloaded at: https://www.mdpi.com/article/10.3390/molecules31213987/s1.

## Abbreviations

AI	Artificial intelligence
DaRT	Diffusing alpha-emitter radiation therapy
DGA	Diglycolamide-based extraction chromatography resin
DNA	Deoxyribonucleic acid
EOB	End of bombardment
ETFE	Ethylene tetrafluoroethylene
FDG	Fluorodeoxyglucose
FAPI	Fibroblast activation protein inhibitor
FMCH	Fluoromethylcholine
HPGe	High-purity germanium
HTO	Tritiated water
ICP-MS	Inductively coupled plasma mass spectrometry
ICP-OES	Inductively coupled plasma optical emission spectrometry
LET	Linear energy transfer
PARP-1	Poly(ADP-ribose) polymerase 1
PET	Positron emission tomography
PRISMA	Preferred Reporting Items for Systematic Reviews and Meta-Analyses
PRISMA-ScR	PRISMA Extension for Scoping Reviews
PSMA	Prostate-specific membrane antigen
PTFE	Polytetrafluoroethylene
QMA	Quaternary methylammonium anion-exchange cartridge
SPECT	Single-photon emission computed tomography
SRIM	Stopping and Range of Ions in Matter
SSTR	Somatostatin receptor
UTEVA	Uranium and tetravalent actinide-selective extraction chromatography resin
