# Supplementary material for: Radionuclidic Impurity Pathways in Cyclotron Production: Insights from 18F and Emerging Theranostic Radionuclides
Source: Molecules. 2026 Nov 5;31(21):3987. doi: 10.3390/molecules31213987 (PMC13765104; doi:10.3390/molecules31213987)
Supplement: Supplementary file 1 [file molecules-31-03987-s001.zip › molecules-4640901-supplementary.pdf]

Supplementary methods and interpretation note[illegible]

Dataset item	Value
Raw records from four Scopus exports	363
Unique publications	318
Duplicate records removed	45
Evidence basis	Title/abstract level
Keywords	Inferred
Open-access status	Source-export flag only

Search source	Raw records
abstract search – isotope therapy	46
abstract search – impurity and cyclotron	190
abstract search – liquid targets	48
abstract search – fluorine	79

This supplementary workbook intentionally contains structured extraction [illegible] should consult the original publications.

s

Interpretation
46 + 190 + 48 + 79 records before deduplication.
Duplicates removed by normalized publication title; source provenance merged.
Repeated articles retained as one record.
Target category, additional radionuclides, and impurity-control information are reported only when stated in title/abstract.
Keywords were inferred from titles/abstracts; they are not author-supplied keywords unless independently identical.
Absence of an open-access flag is not evidence that an article is paywalled.

Unique rows citing source
46
190
48
79

Publicatio

fields rather than copied abstract text. It is intended to document the literature-screeni

Search query / scope

TITLE-ABS-KEY("therap*" AND "radionuclide" AND "cyclotron") AND (LIMIT-TO (SUBJAREA,"MEDI") OR LIMIT-TO (SUBJAREA,"CHEM") OR LIMIT-TO (SUBJAREA,"BIOC") OR LIMIT-TO (SUBJAREA,"PHAR") OR LIMIT-TO (SUBJAREA,"IMMU") OR LIMIT-TO (SUBJAREA,"MULT")) AND (LIMIT-TO (DOCTYPE,"ar")) AND (LIMIT-TO (LANGUAGE,"English")) AND (EXCLUDE (EXACTKEYWORD,"Nonhuman") OR EXCLUDE (EXACTKEYWORD,"Animals") OR EXCLUDE (EXACTKEYWORD,"Animal") OR EXCLUDE (EXACTKEYWORD,"Mice") OR EXCLUDE (EXACTKEYWORD,"Animal Model") OR EXCLUDE (EXACTKEYWORD,"Tissue Distribution") OR EXCLUDE (EXACTKEYWORD,"Prostate Specific Membrane Antigen") OR EXCLUDE (EXACTKEYWORD,"Cell Line, Tumor") OR EXCLUDE (EXACTKEYWORD,"Animal Tissue") OR EXCLUDE (EXACTKEYWORD,"Auger Electron Spectroscopy") OR EXCLUDE (EXACTKEYWORD,"Drug Half Life") OR EXCLUDE (EXACTKEYWORD,"Drug Distribution") OR EXCLUDE (EXACTKEYWORD,"Protein Expression") OR EXCLUDE (EXACTKEYWORD,"Computer Assisted Tomography") OR EXCLUDE (EXACTKEYWORD,"Ablation Therapy")) AND PUBYEAR > 2014 AND PUBYEAR < 2027

TITLE-ABS-KEY ((impurit* OR radionuclid* OR "activation product*") AND (radionuclide* OR radioisotop*) AND cyclotron) AND (EXCLUDE (SUBJAREA , "PHYS") OR EXCLUDE (SUBJAREA , "ENER") OR EXCLUDE (SUBJAREA , "ENVI") OR EXCLUDE (SUBJAREA , "ENGI") OR EXCLUDE (SUBJAREA , "MATH") OR EXCLUDE (SUBJAREA , "AGRI") OR EXCLUDE (SUBJAREA , "EART") OR EXCLUDE (SUBJAREA , "SOCI") OR EXCLUDE (SUBJAREA , "BUSI") OR EXCLUDE (SUBJAREA , "NEUR")) AND (LIMIT-TO (LANGUAGE , "English")) AND (LIMIT-TO (SRCTYPE , "j")) AND (EXCLUDE (DOCTYPE , "re") OR EXCLUDE (DOCTYPE , "ed") OR EXCLUDE (DOCTYPE , "sh") OR EXCLUDE (DOCTYPE , "er") OR EXCLUDE (DOCTYPE , "no") OR EXCLUDE (DOCTYPE , "le")) AND PUBYEAR > 2015

TITLE-ABS-KEY (("Liquid target" OR "solution target") AND cyclotron) AND (LIMIT-TO (DOCTYPE , "ar")) AND (LIMIT-TO (LANGUAGE , "English")) AND PUBYEAR > 2014 AND

TITLE-ABS-KEY ((impurit* OR "activation product*") AND (F-18 OR 18F)) AND (LIMIT-TO (SUBJAREA , "MEDI") OR LIMIT-TO (SUBJAREA , "BIOC") OR LIMIT-TO (SUBJAREA , "PHAR") OR LIMIT-TO (SUBJAREA , "CHEM") OR LIMIT-TO (SUBJAREA , "MULT") OR LIMIT-TO (SUBJAREA , "CENG") OR LIMIT-TO (SUBJAREA , "MATE") OR LIMIT-TO (SUBJAREA , "PHYS")) AND (LIMIT-TO (DOCTYPE , "ar")) AND (LIMIT-TO (LANGUAGE , "English")) AND PUBYEAR > 2014 AND PUBYEAR < 2027

n-ready note

ng dataset and the information available at title/abstract level. For detailed production parameters, separation pr

ocedures, and quantitative results, readers
